# FL-BC-IDS: Evidence-Native Privacy-Aware Hierarchical Federated Intrusion Detection for the Internet of Vehicles

**DOI:** 10.3390/s26175400

**Published:** 2026-08-26

**Authors:** Wisam Makki Alwash, Weam Husham Aljabbari, Muhammed Ali Aydin, Hasan Hüseyin Balik

**Affiliations:** 1 Department of Computer Engineering, Faculty of Electrical and Electronics Engineering, Yildiz Technical University, Istanbul 34220, Türkiye; wisam.alwash@std.yildiz.edu.tr (W.M.A.); weam.jabbari@std.yildiz.edu.tr (W.H.A.); 2 College of Law, University of Babylon, Hillah 51002, Babylon, Iraq; 3 Department of Computer Engineering, Faculty of Engineering, Istanbul University-Cerrahpasa, Avcılar, Istanbul 34320, Türkiye; aydinali@iuc.edu.tr; 4 Department of Computer Engineering, Faculty of Engineering, Istanbul Atlas University, Istanbul 34403, Türkiye

**Keywords:** Internet of Vehicles, intrusion detection, federated learning, differential privacy, blockchain, verifiable evidence

## Abstract

Internet of Vehicles (IoV) intrusion detection systems (IDSs) require collaborative learning that preserves raw-data locality while producing independently checkable post-run evidence. This paper presents FL-BC-IDS, an evidence-native, privacy-aware hierarchical federated IDS in which vehicles train Differentially Private XGBoost models, roadside units perform deterministic admission and tree-bagging aggregation, and the GLOBAL stage forms an equal-weight ensemble over validated RSU models. Signed reports, privacy records, SHA-256/Poseidon commitments, scoped Groth16 proofs, reconstructable public inputs, and digest-pinned blockchain receipts provide a unified verification path. Across 10 seed-controlled runs, the mean ± SD accuracy/F1 values were 0.998021±0.000246/0.983597±0.002053 on CSE-CIC-IDS2018 and 0.999867±0.000152/0.999495±0.000579 on CICIoV2024. With thresholds fixed exclusively from development data, the strict held-out-attack macro recall was 0.8031 and 0.9090 on CSE-CIC-IDS2018 and CICIoV2024, respectively, indicating residual attack-specific generalization limitations; supervised rolling-origin temporal refresh on CSE-CIC-IDS2018 achieved 0.984788 pooled seen-attack recall at a 0.005700 test FPR. A controlled 20-vehicle, eight-round heterogeneity and participation stress test retained 0.998151 accuracy and 0.984782 F1-score. Verification rejected invalid or context-mismatched artifacts and independently checked model–anchor consistency, RSU aggregation replay, commitments, and public inputs. The reported DP budgets are conditional learner-stage bounds for learner-input record instances, not end-to-end guarantees for original pre-preprocessing records.

## 1. Introduction

Connected and software-defined vehicles enable advanced safety, efficiency, and mobility services, but they also expand the cyber–physical attack surface of transportation systems. In Internet of Vehicles (IoV) environments, vehicles, sensors, roadside units (RSUs), users, and backend services interact through heterogeneous vehicle-to-everything (V2X) communication links, while compromise of in-vehicle components, falsified messages, availability attacks, and cross-layer weaknesses can undermine safety-critical decisions and mobility services [1,2]. These characteristics motivate intrusion-detection frameworks that can operate across organizations while preserving detection capability, privacy, and independently checkable security evidence.

This connectivity delivers clear operational benefits, but it also makes security assurance a cross-operator problem rather than a single-owner problem. Security monitoring in IoV is complicated by the fact that data and decisions are distributed across vehicles, fleet operators, roadside infrastructure operators, and backend service providers. Stakeholders may be unwilling or unable to share raw telemetry because of privacy, governance, and competitive constraints, yet they still require assurance that collaborative security mechanisms were executed correctly and that reported outputs were not altered after execution.

Intrusion detection systems (IDSs) are an important defense layer in IoV because they support detection of anomalous or malicious behavior across distributed entities [3]. Centralized collection of vehicular telemetry can nevertheless increase exposure and complicate data governance across independently operated participants [4]. Federated learning (FL) provides an alternative by enabling collaborative model training while retaining raw data at participating vehicles or edge nodes [5]. Blockchain-assisted FL has subsequently been investigated in vehicular environments to strengthen coordination, accountability, traceability, privacy, and participant trust [6,7].

Accordingly, collaborative learning in IoV must satisfy more than predictive utility alone. Shared learning outputs can expose information through inference or reconstruction attacks [8,9]; participation and aggregation must also remain accountable when contributions are distributed across multiple administrative domains [4,10]. FL-BC-IDS therefore treats privacy evidence, participation evidence, deterministic aggregation bindings, verification outcomes, and publication receipts as coupled workflow properties rather than independent add-ons.

Despite growing interest in FL-based IoV IDSs, including blockchain-assisted variants, many pipelines still do not provide a clear and independently checkable record of who contributed to training, which updates were admitted or excluded, how privacy-related settings were bound to the run, and whether published verification results correspond to the same committed inputs. As discussed in Section 2, the representative works reviewed in this paper provide important partial mechanisms; however, they do not combine declared privacy evidence, identity-bound admission, canonical exported artifacts, independent auditor recomputation, scoped proof verification, and receipt-grade on-chain anchoring within a single end-to-end evidence workflow. This weakens accountability across administrative domains even when the IDS itself achieves strong detection results.

This paper addresses that gap by proposing the Federated Learning–Blockchain Intrusion Detection System (FL-BC-IDS), an evidence-native privacy-aware hierarchical federated intrusion-detection framework for IoV. Vehicles train local differential privacy (DP)-enabled Extreme Gradient Boosting (XGBoost) models through Differentially Private XGBoost (DP-XGBoost), RSUs perform deterministic admission and XGBoost tree-based federated aggregation, and the GLOBAL stage combines validated RSU prediction scores into a run-level IDS ensemble. Around this learning workflow, the framework exports canonical, hashed, signed, and committed artifacts so that participation outcomes, declared DP records, aggregation inputs, proof artifacts, public inputs, and blockchain publication receipts remain checkable after execution.

In the proposed framework, blockchain is used as a public-verification and receipt-publication layer with a deliberately limited on-chain role. The on-chain stage records whether a submitted proof/public-input bundle was accepted and stores fixed-length digest pins that identify the corresponding exported artifact set. This provides timestamped anti-substitution evidence that a specific verification result was published for specific committed artifacts, while learning, aggregation, and the main evidence artifacts remain off-chain. The verification scope is intentionally bounded: the framework checks artifact integrity, declared privacy-record binding, participation evidence, deterministic model–anchor consistency, recorded RSU model-transition replay, commitment recomputation, proof/public-input agreement, and receipt consistency, but it does not prove honest local training, raw-data provenance, runtime differential-privacy correctness, benign updates, or full semantic correctness of learning.

The empirical evaluation uses the Communications Security Establishment–Canadian Institute for Cybersecurity Intrusion Detection System 2018 dataset (CSE-CIC-IDS2018) [11] and the Canadian Institute for Cybersecurity Internet of Vehicles 2024 dataset (CICIoV2024) [12] to cover complementary external network-flow and internal Controller Area Network (CAN)-bus attack surfaces under controlled benchmark conditions. The central research question is how a hierarchical FL-based IoV IDS can preserve practical detection utility while making participation, privacy-record binding, aggregation evidence, proof outcomes, and receipt publication independently checkable after execution.

The main contributions are as follows:

- A hierarchical FL-based IoV IDS workflow in which vehicles train locally, RSUs perform deterministic admission and XGBoost tree-based aggregation, and the GLOBAL layer forms an equal-weight prediction ensemble over validated RSU models without centralizing raw telemetry.
- A privacy and participation evidence workflow that binds learner-stage DP-accounting records, model-update digests, canonical report bytes, Ed25519 signatures, and public-key-derived participant identifiers into retained artifacts without claiming end-to-end original-record differential privacy.
- A verifier-oriented evidence path based on canonical records, deterministic model–anchor consistency reconstruction, replayable RSU model transitions, Secure Hash Algorithm 256-bit (SHA-256) and Poseidon commitments, scoped Groth16 proof artifacts, reconstructable public inputs, and digest-pinned blockchain receipts.
- An empirical evaluation covering IDS utility; privacy–utility trade-offs; controlled evidence failures; commitment and public-input reconstruction; proof/publication outcomes; runtime overhead; 20-round operation; controlled federated stress under joint local-data-volume, label, and feature heterogeneity with persistent, isolated, and burst contribution unavailability; stale-round and cross-RSU context challenges; expanded-topology evidence-workflow scaling; and targeted generalization stress tests spanning exact predictor-pattern disjointness, dataset-provided grouping, supervised rolling-origin temporal refresh with development-only operating-point calibration, strict predictor-hash-disjoint leave-one-attack-out detection, and multiclass attack recognition.

The remainder of this paper is organized as follows. Section 2 reviews the most directly related federated, hierarchical, blockchain-assisted, and verifiable IDS research. Section 3 presents the system and threat model, FL-BC-IDS framework, evidence and verification workflow, prototype implementation, and experimental setup. Section 4 reports and discusses IDS performance, dataset-generalization stress tests, privacy–utility and baseline comparisons, 20-round operation, controlled federated heterogeneity and participation stress, evidence verification and blockchain-publication outcomes, runtime overhead, and expanded-topology evidence-workflow scaling. This paper then summarizes the principal limitations and directions for future work before concluding with the main findings and contributions.

## 2. Related Work

This section focuses on the literature most directly related to the research contribution of FL-BC-IDS. Rather than providing a broad taxonomy of IoV attacks or a tutorial treatment of the individual security primitives used by the implementation, this discussion concentrates on federated and hierarchical intrusion detection for vehicular environments and on blockchain-assisted or verifiable federated IDS frameworks that provide the closest methodological comparison.

### 2.1. Federated and Hierarchical IDS for IoV

Automotive and IoV intrusion detection must operate across heterogeneous communication and in-vehicle attack surfaces while respecting latency, data-governance, and distributed-ownership constraints [1,3]. Federated learning addresses part of this problem by allowing participants to contribute model-side information without centralizing their original feature–label records [4,5]. Hierarchical and fog-assisted federated IDS architectures further distribute coordination and aggregation across intermediate edge tiers, which is particularly relevant to vehicle–RSU–server topologies [13]. Tree-based ensemble models have also demonstrated strong applicability to tabular IoV intrusion-detection tasks [14], while CAN-bus-specific IDS research confirms the importance of the internal vehicular attack surface considered in this study [15].

Federation alone does not remove security and privacy risks. Shared learning outputs can remain vulnerable to inference and reconstruction [8,9], and malicious or faulty contributions motivate filtering and aggregation defenses [16]. Recent vehicular FL research has consequently incorporated privacy mechanisms, participant reputation, and blockchain-supported coordination [6,7]. Data heterogeneity remains an additional challenge for federated IDS and motivates partition-aware or knowledge-distillation approaches under non-identically distributed data [17]. These studies establish the need for distributed and privacy-aware IDS operation, but they do not by themselves provide the post-run evidence path targeted by FL-BC-IDS.

### 2.2. Blockchain-Assisted and Verifiable Federated IDSs

The closest prior systems combine blockchain with federated learning to strengthen coordination, trust, privacy, model handling, and intrusion detection in vehicular environments. Liu et al. use masked uploads, RSU pre-aggregation, and trust-driven model-chain consensus [18]; Abdel-Basset et al. combine blockchain-managed federated intrusion detection with gradient screening [19]; Lv et al. integrate differential privacy and blockchain-supported misbehavior detection [20]; and Jiang et al. combine adaptive differential privacy with blockchain-integrated update screening [21]. More recent systems address model/log recording, reputation management, model selection, and privacy-preserving aggregation [22,23,24,25].

Verifiability is addressed more directly by Smahi et al., who combine blockchain-based federated learning with zero-knowledge succinct non-interactive argument of knowledge (zk-SNARK)-style verification of selected local-model claims in V2X environments [26]. This establishes an important proof-oriented precedent. The research question addressed by FL-BC-IDS is nevertheless different: whether a completed hierarchical federated IDS run can retain one reconstructable evidence path linking participant admission, declared privacy records, canonical contribution artifacts, deterministic commitments, proof/public-input agreement, and digest-pinned public receipts.

Table 1 highlights a clear distinction between FL-BC-IDS and the representative blockchain-assisted and verifiable federated IDS frameworks considered in this study. Prior approaches provide valuable mechanisms for privacy preservation, trust management, blockchain coordination, malicious-update handling, model or log recording, and selected proof-oriented verification. FL-BC-IDS extends this landscape by integrating declared privacy evidence, identity-bound admission, canonical exported artifacts, independent auditor recomputation, scoped Groth16 verification, and digest-bound public receipt anchoring into a unified end-to-end evidence workflow for hierarchical federated intrusion detection.

Table 1 uses ✓, ∼, and ✗ to denote explicit reproducible support, partial or indirect support, and absence or non-reporting of the corresponding dimension, respectively. The columns distinguish declared privacy evidence, identity-bound admission evidence, deterministic exported artifacts, independent auditor recomputation, zero-knowledge (ZK) or verifiable-computation support, and receipt-grade on-chain confirmation bound to specific artifact digests.

The comparison demonstrates that FL-BC-IDS complements the strengths of existing privacy-, trust-, blockchain-, and proof-oriented approaches with a substantially more integrated evidence path. Its distinguishing contribution is the preservation of canonical, identity-bound, privacy-bound, digest-bound, proof-linked, and receipt-pinned artifacts that allow an independent auditor to reconstruct and verify the security-relevant evidence history of a completed hierarchical federated IDS run.

## 3. Materials and Methods

This section presents the methodological design, implementation, and evaluation setup of FL-BC-IDS.

### 3.1. System Model and Threat Scope

This subsection defines the system entities, trust assumptions, threat scope, and verification boundaries of FL-BC-IDS.

#### 3.1.1. System Entities and Hierarchy

FL-BC-IDS is organized as a hierarchical federated IDS workflow with three operational tiers: vehicles, RSUs, and a GLOBAL aggregation and verification stage. Vehicles retain local intrusion-detection data and perform local DP-XGBoost training without exporting raw telemetry. Each RSU coordinates its assigned vehicles, verifies submitted participation evidence, admits or excludes contributions under deterministic checks, and constructs an RSU-level XGBoost model through federated tree bagging. After the configured RSU federated rounds are completed, the GLOBAL stage validates the eligible RSU bundles and combines the prediction scores of the validated RSU models into a run-level IDS ensemble while exporting GLOBAL-scope evidence for verification and publication. The hierarchy therefore uses model-space aggregation at the RSU tier and decision-space aggregation at the GLOBAL tier.

The learning workflow is supported by an evidence workflow. At the vehicle tier, each participant produces local learning outputs together with declared DP records, canonical report bytes, digest bindings, and Ed25519 signatures. At the RSU tier, accepted participant records are ordered deterministically and committed through SHA-256 and Poseidon Merkle roots. At the GLOBAL stage, RSU outputs are bound into GLOBAL manifests and proof/public-input artifacts. The blockchain layer is not part of training or aggregation; it is used only to publish accepted verification outcomes and fixed-length digest pins.

#### 3.1.2. Threat Scope and Verification Boundaries

The framework assumes that raw telemetry remains local to vehicles and is not disclosed to RSUs, the GLOBAL stage, or the blockchain layer. The evaluated threat scope includes invalid participation signatures, mismatched DP-record bindings, corrupted exported evidence, artifact substitution, commitment inconsistency, public-input mismatch, and disagreement between off-chain verification artifacts and on-chain receipts.

Adversaries may control one or more vehicles and may submit arbitrary model updates together with malformed metadata, replayed reports, collusive submissions, or scope-mismatched evidence. FL-BC-IDS verifies the identity, context, and retained evidence associated with a submitted update, but it does not determine whether the model update itself is poisoned, backdoored, or otherwise semantically malicious. Network adversaries may delay, drop, replay, or reorder messages, and exported artifacts may be inspected after the run. RSUs and the GLOBAL stage are treated as operationally powerful stages because they control admission, inclusion, ordering, aggregation, and exported manifests. FL-BC-IDS therefore does not assume that every operational decision is fair; instead, it makes selected decisions externally reviewable through explicit admission outcomes, inclusion masks, exclusion reasons, canonical ordering rules, commitments, proof artifacts, and receipt bindings. Because each signed vehicle report binds the federated-round identifier and RSU scope, reuse of that report in a different round or RSU context produces a context mismatch and is rejected by the admission checks. This context binding does not constitute a general Byzantine-robustness or anti-replay guarantee for all protocol states.

The framework assumes correct participant-key provisioning and correct deployment of the blockchain verifier contracts. Key provisioning or revocation failures, participant-key compromise, endpoint compromise, and contract misdeployment remain outside the guaranteed boundary.

FL-BC-IDS complements conventional authenticated transport rather than replacing it; its evidence guarantees operate at the artifact level through canonical bytes, hashes, signatures, commitments, proofs, and receipt bindings.

Quantum cryptography, particularly quantum key distribution (QKD), provides an alternative mechanism for establishing shared cryptographic keys [27]. QKD is complementary to FL-BC-IDS because the proposed framework addresses federated intrusion detection and evidence verification rather than quantum key establishment or distribution. Its integration into vehicle–RSU or infrastructure communication therefore remains a separate direction requiring suitable quantum communication infrastructure and dedicated evaluation.

The scope is intentionally bounded. FL-BC-IDS does not prove honest local training, raw-data provenance, cryptographic enforcement of runtime DP execution, or semantic correctness of submitted model updates. It does not provide poisoning or backdoor detection, collusion resistance, Byzantine-robust or malicious-aggregation-resistant learning, complete Sybil prevention, protection after compromise of a participant signing key, endpoint security, or universal IoV security.

Table 2 summarizes the implemented and out-of-scope security and verification properties of FL-BC-IDS. The detailed artifact formats and verification procedures are presented in Section 3.3, and the corresponding outcomes are reported in Section 4.

The symbols ✓ and ✗ indicate implemented support and out-of-scope properties, respectively. This scope definition clarifies that FL-BC-IDS reduces blind trust by making selected failures rejected, detected, or reviewable, but it does not claim universal IoV security.

### 3.2. FL-BC-IDS Framework

This subsection defines the hierarchical learning and evidence architecture of FL-BC-IDS. The learning workflow is complemented by independently checkable privacy, participation, commitment, proof, and publication evidence without claiming full correctness of the underlying learning process.

#### 3.2.1. Run Context and Notation

Let V={1,…,n} denote the run-scope set of vehicles and let R={1,…,s} denote the run-scope set of RSUs. Vehicles are assigned to RSUs through a fixed run-level assignment function ρ

(1) ρ:V→R.

Federated training within each RSU proceeds in rounds t∈{1,…,T}. Each vehicle *i* holds a fixed local training shard $D_{i}$. For RSU *r* in round *t*, the admitted vehicle set is

(2) $$V_{r,t}^{\mathrm{adm}}\subseteq \{i\in V:\rho \left(i\right)=r\}.$$

The serialized model contribution produced by vehicle *i* in round *t* is denoted by $\Delta_{i,t}$, and the aggregated XGBoost booster maintained by RSU *r* after that round is denoted by $B_{r,t}$. At the terminal evaluated round *T*, let $R_{T}^{\mathrm{ok}}\subseteq R$ denote the RSUs whose model and evidence bundles satisfy the GLOBAL inclusion conditions. For an input *x*, $p_{r,T}\left(x\right)$ denotes the prediction score produced by the validated RSU model $B_{r,T}$, and $p_{G}\left(x\right)$ denotes the resulting GLOBAL ensemble score.

#### 3.2.2. End-to-End Protocol Lifecycle

Within each RSU federation, round *t* begins from the current RSU model state $B_{r,t-1}$, when a previous aggregated state is available. Participating vehicles continue local DP-XGBoost training from that state, export their newly added serialized model contributions $\Delta_{i,t}$, and bind the contribution digest, DP-record digest, anchor payload, round context, and identity context into signed participation reports. The RSU verifies candidate reports, determines the admitted contribution set, applies deterministic XGBoost tree bagging, and obtains the next RSU model $B_{r,t}$ together with RSU-scope commitments and proof artifacts. After the configured federated rounds have completed for all RSUs, the GLOBAL stage validates the terminal RSU bundles, records the RSU inclusion mask, combines the prediction scores of the validated RSU models into the run-level ensemble, and exports GLOBAL-scope evidence. The blockchain layer then publishes digest-pinned receipts for the accepted RSU and GLOBAL proof/public-input bundles.

The lifecycle distinguishes participant dropout from invalid evidence. A missing contribution is absent from the admitted set for that round, whereas a received but invalid contribution is explicitly excluded with a recorded reason. The same principle applies to RSU bundles at the GLOBAL stage.

#### 3.2.3. Data Contract and Hierarchical Partitioning

FL-BC-IDS evaluates two complementary IoV attack surfaces. CSE-CIC-IDS2018 represents external network-flow attacks, while CICIoV2024 represents internal CAN-bus attacks [11,12]. Both datasets are converted to the same binary IDS task, BENIGN versus ATTACK. Each run is bound to a fixed dataset identity, ordered feature schema, preprocessing definition, and train/validation/test split.

Only the training split is partitioned across the hierarchical FL topology. Within each seed-specific execution, the validation and test splits remain fixed evaluation artifacts and are not partitioned among vehicles. Preprocessing parameters, including the imputation, scaling, encoding, and numeric bounds used before DP-XGBoost training, are fitted from the training split only and then applied unchanged to the validation and test data. This prevents validation/test information from entering the fitted preprocessing operations, but it does not by itself make the row-level partitions predictor-pattern-disjoint. The complementary generalization analyses reported in Section 4.2 evaluate cross-partition predictor recurrence separately. This separation ensures that model training, threshold selection, testing, artifact generation, and audit interpretation refer to the same reproducible data contract while avoiding conflation of preprocessing leakage with predictor-pattern recurrence.

The partitioning procedure assigns training examples to RSU-local pools and then to vehicle shards under deterministic rules. The principal compact benchmark therefore remains a controlled hierarchical setting rather than an uncontrolled real-world mobility simulation. A separate CSE-CIC-IDS2018 federated stress experiment, described in Section 4.4, evaluates joint local-data-volume, label, and feature heterogeneity together with persistent, isolated, and burst contribution unavailability and signed-context admission challenges. This additional experiment extends the operational evaluation beyond the balanced, stable-participation compact benchmark. Physical vehicle mobility, time-varying RSU association, packet-level wireless behavior, and stateful seamless handoff remain outside the evaluated scope.

#### 3.2.4. Local Learner and Update Contract

Vehicles use an XGBoost-family gradient-boosted decision tree classifier for tabular IDS learning [28]. The prototype orchestrates this learner through the Flower and Sarus DP-XGBoost implementations; the exact software versions are reported in Section 3.4, while the adopted DP-XGBoost construction is described by Grislain and Gonzalvez [29]. At federated round *t*, vehicle *i* assigned to RSU *r* receives the current RSU model state when available and continues DP-XGBoost training on its local shard $D_{i}$. Before local training, the implementation records the current number of boosted rounds. After training, native XGBoost booster slicing extracts the rounds added during the current fit operation, and the resulting booster slice is serialized as the vehicle contribution $\Delta_{i,t}$. Raw feature–label records remain local and are never included in the transmitted contribution.

The conditional learner-stage privacy account applies to the DP-protected tree-learning outputs of the adopted DP-XGBoost mechanism. The native serialized booster contribution is retained as an implementation artifact required by the evaluated Flower aggregation and evidence workflow and is not itself claimed to constitute an independently publishable differentially private release.

The exact serialized contribution bytes are bound to the round context by

(3) $$h_{i,t}^{\Delta }=\mathrm{SHA}256\left(\mathrm{model}\_\mathrm{delta}\_{\mathrm{bytes}}_{i,t}\right).$$

The digest is included in the signed vehicle report and is recomputed during RSU-side verification.

RSU model aggregation is performed with Flower’s XGBoost bagging strategy rather than FedAvg-style numerical parameter averaging. Let

(4) $$\pi_{r,t}=\left(\pi_{r,t}\left(1\right),\ldots ,\pi_{r,t}\left(m_{r,t}\right)\right)$$

denote the deterministic cid-ordered sequence of admitted client results at RSU *r*, where $m_{r,t}=\left|V_{r,t}^{\mathrm{adm}}\right|$. The RSU update can therefore be written as

(5) $$B_{r,t}=A_{\mathrm{RSU}}\left(B_{r,t-1};\Delta_{\pi_{r,t}\left(1\right),t},\ldots ,\Delta_{\pi_{r,t}\left(m_{r,t}\right),t}\right),$$

where $A_{\mathrm{RSU}}$ denotes the deterministic XGBoost tree-bagging operation implemented by FedXgbBagging. The evaluated configuration fixes num_parallel_tree = 1, so tree groups are interpreted under the single-tree-per-parallel-group XGBoost configuration. The serialized tree order within each contribution is retained by the XGBoost representation, while the sequence of admitted client contributions is fixed before aggregation.

Model-tree aggregation is not weighted by the number of local training examples. Client sample counts are used separately for weighted aggregation of the distributed evaluation metrics and do not scale the XGBoost tree contributions themselves. The implementation also performs no content-based tree deduplication: two distinct admitted client–round contributions remain distinct aggregation inputs even if individual tree structures happen to coincide. Contribution identity is instead maintained through the participant, RSU, round, and model-digest context recorded by the evidence workflow.

After RSU aggregation, the returned serialized model is deserialized as an XGBoost Booster and its tree structure is inspected before the latest valid RSU model is retained for persistence and evaluation. The RSU aggregation output is therefore represented and subsequently evaluated as a native XGBoost booster rather than as an external collection of unrelated trees.

#### 3.2.5. Vehicle-Side Privacy and Participation Evidence

Each vehicle exports learning and evidence objects for the current federated round. The learning object is the serialized model contribution produced during that round. The accompanying evidence objects include a canonical differential-privacy (DP) record, its digest binding, a quantized anchor payload, and a public-key-bound signed participation report.

The DP-XGBoost mode used in FL-BC-IDS applies the adopted pure-ϵ mechanisms during vehicle-side tree training.

The adopted realization treats approxDP as the differentially private tree-learning mode of the DP-XGBoost implementation, not as approximate (ϵ,δ)-DP. The privacy mechanism is treated as a Laplace-based pure-ϵ construction with DP histogram support, exponential-mechanism split selection, and Laplace-perturbed leaf values [29,30,31].

For each round, the vehicle persists a canonical DP record and binds it by

(6) $$h_{i,t}^{DP}=\mathrm{SHA}256\left(\mathrm{dp}\_\mathrm{record}\_{\mathrm{bytes}}_{i,t}\right).$$

This digest makes the declared DP evidence tamper-evident, but it does not cryptographically prove that DP training was executed correctly at runtime.

The reported privacy accountant is defined at the record level on the learner-input multiset supplied to DP-XGBoost after fitted transformation and any train-only oversampling. For vehicle *i*, two local learner-input datasets $D_{i}$ and $D_{i}^{\prime }$ are neighboring when they differ by one such record instance while all other vehicle shards remain unchanged. The resulting guarantee is therefore a conditional learner-stage record-level guarantee rather than vehicle-level or end-to-end original-record differential privacy.

The evaluated benchmark preprocessing contains data-dependent operations outside this learner-stage account, including training-derived imputation and scaling parameters, encoding parameters when applicable, and feature-domain bounds. In addition, RandomOverSampler may map one original training record to multiple learner-input instances before vehicle partitioning. Consequently, the numerical learner-stage budget cannot be transferred unchanged to an original pre-preprocessing record.

The binary labels satisfy

(7) y∈{0,1}⊂[−1,1],

which is consistent with the bounded-label DP-XGBoost construction. For the squared-error learner, gradient-data filtering bounds the per-record gradient magnitude by

(8) $$g_{\ell }^{*}=1.$$

The corresponding split-gain sensitivity is

(9) $$\Delta G=3{\left(g_{\ell }^{*}\right)}^{2}=3,$$

and the DP-XGBoost leaf-value sensitivity satisfies

(10) $$\Delta V\leq \frac{2g_{\ell }^{*}}{N_{\min}+1+\lambda }.$$

Because the evaluated learner uses objective=reg:squarederror, the per-record Hessian is one; therefore, min_child_weight = 500 corresponds to $N_{\min}=500$ for this construction. With λ=1, substituting $g_{\ell }^{*}=1$ from Equation (8) and $N_{\min}=500$ into Equation (10) gives

(11) $$\Delta V\leq \frac{2}{500+1+1}=\frac{2}{502}\approx 0.003984.$$

These sensitivities are handled internally by the DP-XGBoost histogram, exponential-mechanism split-selection, and Laplace-perturbed leaf-value construction [29,31].

For every transformed feature *j*, the implementation computes run-level bounds

(12) $$L_{j}=\min_{x\in D_{\mathrm{train}}}x_{j},\;U_{j}=\max_{x\in D_{\mathrm{train}}}x_{j},$$

from the preprocessed benchmark training matrix before hierarchical vehicle partitioning. Each vehicle-side transformed training value is bounded coordinate-wise according to

(13) $${\tilde{x}}_{j}=\min\left\{U_{j},\max\left\{L_{j},x_{j}\right\}\right\}.$$

The same bound vectors are supplied through the feature_min and feature_max arguments of every vehicle-side DP-XGBoost DMatrix. Validation and test representations are likewise constrained to the same run-level bounds. No separate federated-learning update-norm or $\ell_{1}$-clipping mechanism is used.

The feature bounds are computed once from the benchmark training data and then held fixed throughout the federated run. Consequently, the privacy values reported below characterize the DP-XGBoost learner conditional on the fitted preprocessing artifact. They do not include a privacy cost for obtaining the feature bounds or the other training-derived preprocessing quantities.

Let $\left(\epsilon_{\mathrm{pre}},\delta_{\mathrm{pre}}\right)$ denote the privacy cost of obtaining all data-dependent preprocessing quantities used by the protected learning path, including imputation, scaling, encoding parameters when applicable, and feature-domain bounds. If these quantities are public or externally fixed independently of the protected records, then $\epsilon_{\mathrm{pre}}=\delta_{\mathrm{pre}}=0$. If they are estimated through differentially private mechanisms, their privacy cost must instead be composed with the learner-stage cost.

Let

(14) $$\epsilon_{\mathrm{tree}}=0.25$$

denote the pure-DP budget assigned to one DP-XGBoost tree, and let

(15) q=0.2

denote the random row-subsampling fraction. Under the privacy-amplification-by-subsampling rule used by the adopted DP-XGBoost accountant, for sampling without replacement, the effective privacy cost of one tree is

(16) $$\epsilon_{\mathrm{amp}}=\log\left[1+q\left(e^{\epsilon_{\mathrm{tree}}}-1\right)\right].$$

Substituting $\epsilon_{\mathrm{tree}}$ and *q* from Equations (14) and (15) into Equation (16) gives

(17) $$\epsilon_{\mathrm{amp}}=\log\left[1+0.2\left(e^{0.25}-1\right)\right]=0.0552502843.$$

Each participating vehicle performs 10 local boosting iterations per federated round with num_parallel_tree = 1. Thus, at most, 10 new trees are added during one complete local fit. Using the amplified per-tree privacy cost in Equation (17), the basic sequential composition of these pure-ϵ mechanisms gives

(18) $$\epsilon_{\mathrm{round}}\leq 10\left(0.0552502843\right)=0.5525028434\approx 0.5525.$$

The same vehicle shard participates in two federated rounds in the compact experiments. Applying sequential composition across those two rounds to the per-round bound in Equation (18) therefore gives

(19) $$\epsilon_{\mathrm{total}}\leq 2\left(0.5525028434\right)=1.1050056867\approx 1.1050.$$

For the conditional learner-stage account, the number of vehicles does not by itself introduce an additional multiplicative factor for distinct learner-input record instances. After preprocessing and oversampling, each such instance is assigned to one vehicle shard. Mechanisms operating on disjoint vehicle shards therefore compose in parallel with respect to these learner-input instances, so the four-vehicle compact configuration retains the learner-stage bound $\epsilon_{\mathrm{total}}\leq 1.1050$ rather than multiplying it by four.

The corresponding end-to-end original-record requirement can be stated separately. Let *c* denote a guaranteed upper bound on the number of learner-input instances attributable to one original protected training record under the preprocessing/resampling contract, and let $\epsilon_{\mathrm{learn}}$ denote the applicable composed learner-stage privacy bound. Because the evaluated DP-XGBoost learner uses pure ϵ-DP, preprocessing-aware composition gives

(20) $$\epsilon_{\mathrm{raw}}\leq \epsilon_{\mathrm{pre}}+c\;\epsilon_{\mathrm{learn}},$$

with

(21) $$\delta_{\mathrm{raw}}\leq \delta_{\mathrm{pre}}.$$

For the compact two-round configuration, $\epsilon_{\mathrm{learn}}\leq 1.1050056867$.

Under an end-to-end contract in which all fitted preprocessing quantities are public or externally fixed and one original record maps to at most one learner-input instance, $\epsilon_{\mathrm{pre}}=\delta_{\mathrm{pre}}=0$ and c=1; the compact bound would then reduce to $\epsilon_{\mathrm{raw}}\leq 1.1050$ with $\delta_{\mathrm{raw}}=0$. Alternatively, differentially private preprocessing contributes its own $\left(\epsilon_{\mathrm{pre}},\delta_{\mathrm{pre}}\right)$ cost, and bounded record replication contributes through *c* [31].

The completed benchmark runs used training-derived preprocessing parameters and train-only random oversampling without a separately accounted DP preprocessing budget or a guaranteed original-record multiplicity cap. Therefore, no numerical $\epsilon_{\mathrm{raw}}$ is retrospectively claimed for those runs; the reported numerical values remain conditional learner-stage bounds for learner-input record instances.

The accountant uses pure ϵ-DP with δ=0 and basic composition. No advanced-composition, Rényi-DP, Gaussian-DP, moments, or related alternative accountant is applied. The calculation also gives no privacy credit for possible early stopping and is therefore reported as an analytical upper bound [29,31].

Each vehicle also computes a fixed-length quantized anchor payload $q_{i,t}\in Z^{M}$ from the shared run-level anchor set. The anchor payload is not a privacy mechanism and is not treated as a substitute for the serialized XGBoost contribution. Instead, it provides a deterministic model-side consistency witness.

For independent verification, the verifier uses the exact retained pre-update model state together with the exact serialized contribution $\Delta_{i,t}$ to reconstruct the corresponding post-update vehicle model under the frozen XGBoost model contract. The reconstructed model is evaluated on the same fixed anchor set and subjected to the same clipping and fixed-point quantization rule. Successful model-linkage verification requires exact equality between this independently reconstructed anchor and the submitted anchor payload.

Its canonical bytes are bound by

(22) $$h_{i,t}^{q}=\mathrm{SHA}256\left(q\_{\mathrm{bytes}}_{i,t}\right).$$

Let $B_{i,t}^{\mathrm{pre}}$ denote the exact model state from which the vehicle fit begins and let ${\tilde{B}}_{i,t}$ denote the independently reconstructed post-update model. The verifier computes

(23) $${\tilde{B}}_{i,t}=A_{\mathrm{local}}\left(B_{i,t}^{\mathrm{pre}},\Delta_{i,t}\right),$$

followed by

(24) $$q_{i,t}^{*}=Q\left(f_{{\tilde{B}}_{i,t}}\left(X_{A}\right)\right),$$

where $X_{A}$ is the fixed run-level anchor matrix and Q denotes the declared clipping and fixed-point quantization rule. Model–anchor consistency requires

(25) $$q_{i,t}^{*}=q_{i,t}\;\mathrm{and}\;\mathrm{SHA}256\left(q\_{\mathrm{bytes}}_{i,t}^{*}\right)=h_{i,t}^{q}.$$

The exact serialized contribution must additionally reproduce $h_{i,t}^{\Delta }$ from Equation (3). Consequently, signing an unrelated model-update digest and anchor digest does not satisfy the complete model-linkage verification.

Finally, the vehicle signs a canonical report containing the round identifier, RSU scope, update digest, DP-record digest, anchor digest, and participant-identity context. The report is signed with an Ed25519 key, and the verifier derives or checks the stable participant identifier from the verified public key [32].

#### 3.2.6. RSU Admission and Edge Aggregation

Each RSU verifies candidate vehicle contributions before aggregation. A contribution is admitted only if the RSU can verify the signed report, confirm the declared round and RSU scope, check that the serialized update matches the reported update digest, confirm that the DP-record digest is well formed and consistently bound, and verify that the delivered anchor payload matches its digest. For post-run evidence verification, the retained contribution is additionally subject to independent model-linkage verification according to Equations (23)–(25). Thus, successful evidence verification requires not only independent digest agreement for the model and anchor artifacts but also deterministic agreement between the retained model transition and the submitted anchor payload.

The RSU applies a two-stage deterministic ordering rule. Evidence-valid candidate contributions are first ordered lexicographically by (vehicle_id,did,cid) for deterministic capacity selection. Here, did denotes the implementation field containing the stable public-key-derived participant identifier. If the number of otherwise-valid candidates exceeds the configured proof capacity, the first entries under this order are retained and the remaining candidates are recorded as capacity exclusions. After the admitted set has been fixed, the corresponding Flower client results are sorted lexicographically by cid, yielding the sequence $\pi_{r,t}$ defined in Equation (4), before XGBoost tree aggregation. The same cid-ordered admitted set is recorded in the RSU manifest and used to construct the canonical client-envelope sequence underlying the SHA-256 and Poseidon commitments.

The RSU then applies the aggregation operator $A_{\mathrm{RSU}}$ defined in Equation (5) to obtain $B_{r,t}$. The exported RSU round bundle records the admitted and excluded participants, the RSU aggregation context, the SHA-256 and Poseidon Merkle roots, and the RSU-scoped proof/public-input artifacts. If no vehicle satisfies the required admission conditions, the corresponding verification scope is marked invalid rather than silently producing a successful verification result.

#### 3.2.7. Global Aggregation and Receipt Publication

After the RSU federated training stage, the GLOBAL stage validates the terminal RSU bundles under the declared run configuration. It checks the required artifact fields, ordering consistency, manifest bindings, commitment values, and RSU-scoped proof outcomes. RSUs are represented in ascending rsu_id order, and the GLOBAL manifest records an inclusion mask aligned with that order. Only validated RSU models satisfying the configured inclusion conditions participate in the run-level prediction ensemble.

Let $K_{T}=\left|R_{T}^{\mathrm{ok}}\right|>0$, and let $p_{r,T}\left(x\right)$ denote the prediction score produced by the saved terminal model of included RSU *r*. The GLOBAL prediction score is defined as the coordinate-wise clipped mean

(26) $$p_{G}\left(x\right)=\frac{1}{K_{T}}\sum_{r\in R_{T}^{\mathrm{ok}}}\mathrm{clip}\left(p_{r,T}\left(x\right),0,1\right).$$

All included RSUs therefore receive equal predictive weight; the number of local training examples associated with an RSU does not enter the GLOBAL fusion coefficient. The GLOBAL decision rule is

(27) $${\hat{y}}_{G}\left(x\right)=1\left[p_{G}\left(x\right)\geq \tau_{G}\right],$$

where $\tau_{G}$ is selected on the fixed validation split by maximizing the F1-score and is subsequently held fixed for test evaluation.

The GLOBAL operation is therefore a decision-level ensemble of independently valid RSU XGBoost models rather than a second tree-concatenation step. FL-BC-IDS does not require the RSU trees to be rewritten into one monolithic XGBoost booster: the run-level predictor is the explicitly defined ensemble $p_{G}$, whose component models remain native loadable XGBoost boosters. If no RSU satisfies the inclusion conditions, no GLOBAL prediction ensemble is produced for that scope.

The blockchain layer operates only after the relevant off-chain artifacts have been generated. It verifies the submitted Groth16 proof against the supplied public inputs and records a compact receipt containing the verification outcome and fixed-length digest pins to the corresponding exported artifact set [33,34]. The blockchain does not train models, aggregate updates, store raw telemetry, store per-client records, or replace off-chain auditor reconstruction.

### 3.3. Evidence and Verification Workflow

This subsection defines the evidence workflow that makes a completed FL-BC-IDS run independently checkable. The workflow binds local contributions, DP records, participation reports, commitment roots, proof artifacts, public inputs, and blockchain receipts into a consistent post-run evidence path. The verification target is intentionally bounded: FL-BC-IDS checks whether exported artifacts, reconstructed model–anchor relations, recorded RSU model transitions, and scoped proof/public-input records agree with the declared run context. It does not prove honest local training, raw-data provenance, correct runtime DP execution, benign model updates, or full semantic correctness of the learned IDS model.

#### 3.3.1. Canonical Artifacts and Digest Binding

FL-BC-IDS uses canonical encodings so that the same logical artifact always produces the same byte sequence before hashing, signing, commitment construction, proof-input generation, or receipt publication. This is necessary because the verification pipeline operates over bytes rather than informal semantic equivalence. Without canonical encoding, two representations of the same logical record could produce different digests, signatures, commitments, and public-input bindings [35].

For each persisted artifact *x*, the basic integrity rule is

(28) $$h_{x}=\mathrm{SHA}256\left(\mathrm{canonical}\_\mathrm{bytes}\left(x\right)\right).$$

Auditors later recompute the digest over the exact persisted bytes and compare it with the recorded value. This rule applies to model-delta bytes, DP records, anchor payloads, vehicle reports, manifests, proof artifacts, and receipt-binding files.

#### 3.3.2. Signed Vehicle Reports and DP-Record Evidence

The vehicle report is the first evidence object that binds learning and accountability together. It records the participant identity context, federated round, RSU scope, model-update digest, DP-record digest, and anchor digest. The report is serialized canonically and signed using Ed25519. At RSU admission time, the RSU verifies the signature, derives or checks the public-key-based participant identifier, and confirms that all declared digests match the delivered objects.

The DP record is treated as declared privacy evidence. Its digest is carried through the signed report and downstream commitments so that later substitution or mismatch can be detected. However, the DP record is not treated as a cryptographic proof of correct DP execution. This limitation is important because the verification workflow proves artifact consistency and binding, not runtime compliance of the DP-XGBoost implementation.

#### 3.3.3. Merkle Commitments and Scoped Proof Context

At the RSU scope, FL-BC-IDS builds two complementary commitment roots over the same canonically ordered admitted contribution envelopes. The SHA-256 Merkle root supports conventional off-chain audit recomputation, while the Poseidon Merkle root supports efficient binding inside the arithmetic circuit used by the Groth16 proof [36,37,38]. These roots are complementary, not interchangeable: SHA-256 is used for ordinary artifact integrity, while Poseidon is used for proof-friendly field arithmetic.

At the GLOBAL scope, the authoritative binding object is the canonical GLOBAL manifest. It records the ordered RSU identifiers, the aligned inclusion mask, and the RSU round roots used by the server. This manifest prevents ambiguity about which RSUs were included in the GLOBAL evidence scope and prediction ensemble and which committed RSU contexts were referenced by the GLOBAL proof.

The scoped proof context is therefore defined by the declared scope identifier, ordered participant or RSU list, inclusion/admission mask, commitment roots, anchor aggregation values, and public inputs. If any artifact, ordering decision, digest field, commitment root, inclusion decision, or public-input value changes, independent reconstruction will expose the mismatch.

#### 3.3.4. Groth16 Anchor-Aggregation-Consistency Verification

The Groth16 layer verifies only a scoped anchor-aggregation-consistency statement over declared public inputs and private witness values [33,34,38]. At the RSU scope, the statement checks that the reported masked aggregation over admitted vehicle anchor payloads is consistent with the declared RSU context. At the GLOBAL scope, the statement checks the corresponding masked aggregation over included RSU-level anchor aggregates.

The verification workflow separates three complementary relations: signed artifact binding, deterministic model consistency, and scoped arithmetic proof verification. The signed contribution report and committed evidence envelope bind the model-update digest and anchor-payload digest to the same participant, RSU, and round context. The deterministic model-linkage check then reconstructs the post-update model associated with the exact serialized contribution and requires its independently reconstructed quantized anchor to equal the submitted anchor according to Equations (23)–(25). Therefore, co-signing two internally unrelated model and anchor artifacts is insufficient for a successful verification verdict.

RSU model aggregation is verified separately by deterministic replay. For the recorded admitted-client order $\pi_{r,t}$, the independent verifier re-executes the same frozen RSU aggregation operator used by the evaluated implementation:

(29) $${\hat{B}}_{r,t}=A_{\mathrm{RSU}}\left(B_{r,t-1};\Delta_{\pi_{r,t}\left(1\right),t},\ldots ,\Delta_{\pi_{r,t}\left(m_{r,t}\right),t}\right).$$

The reconstructed transition is accepted only when

(30) $$\mathrm{SHA}256\left(\mathrm{model}\_\mathrm{bytes}({\hat{B}}_{r,t})\right)=\mathrm{SHA}256\left(\mathrm{model}\_\mathrm{bytes}(B_{r,t})\right).$$

The replay context retains the preceding model digest, deterministic ordered contribution digests, resulting aggregate-model digest, and model-contract identifier so that modification or reordering of the recorded transition is independently detectable.

Groth16 retains a deliberately narrower role. The circuit verifies the declared anchor-aggregation arithmetic over the admitted proof scope; it does not execute the complete XGBoost program. Model–anchor derivation and the recorded XGBoost aggregation transition are instead checked independently by deterministic reconstruction and replay. Accordingly, successful end-to-end evidence verification requires both these model-level checks and the existing signature, commitment, Groth16, public-input, and receipt checks. These mechanisms do not prove honest local training, genuine raw data, correct runtime DP execution, benign model updates, or semantic robustness of the resulting IDS.

#### 3.3.5. Blockchain Receipts and Auditor Reconstruction

The blockchain receipt records whether a submitted proof is verified for the supplied public inputs and stores fixed-length digest pins to the corresponding off-chain artifact set. This provides a public, timestamped, tamper-evident publication record for the accepted proof/public-input/artifact-binding tuple. The receipt is not a substitute for the off-chain artifacts; it only anchors their accepted verification outcome.

The publication layer can be realized under different append-only trust models. FL-BC-IDS uses a public blockchain to place accepted proof-verification outcomes and artifact-digest pins on an externally maintained, publicly queryable, consensus-replicated record, thereby reducing reliance on a single operational custodian. Blockchain execution remains outside IDS training, aggregation, and inference.

An independent auditor reconstructs the evidence path as follows:

1. Recompute SHA-256 digests over persisted canonical artifacts according to Equation (28);
2. Verify Ed25519 vehicle signatures and participant identity bindings;
3. Verify that each exact serialized model contribution reproduces the model-update digest carried by its signed report;
4. Reconstruct each retained post-update vehicle model, recompute its quantized anchor on the fixed anchor set, and require exact agreement according to Equations (23)–(25);
5. Replay each recorded RSU model transition from the preceding RSU model and exact deterministically ordered admitted contributions according to Equations (29) and (30);
6. Rebuild the SHA-256 Merkle root from admitted contribution envelopes;
7. Rebuild the Poseidon proof root from the proof-oriented committed context;
8. Reconstruct the expected public-input vector for the declared RSU or GLOBAL scope;
9. Verify agreement between exported public inputs, proof artifacts, manifests, and commitment roots;
10. Compare off-chain artifact digests with the digest pins recorded in the blockchain receipt.

If all checks agree, the auditor can conclude that the exported evidence is internally consistent with the declared scope, that the retained model contributions are consistent with the corresponding anchor evidence under the declared reconstruction rule, that the recorded RSU model transition is reproducible from its retained ordered inputs, and that the corresponding proof/public-input bundle was accepted and receipt-pinned. If a check fails, the workflow identifies the mismatch location, whether a corrupted signature, model-delta digest substitution, model–anchor derivation mismatch, aggregation replay mismatch, mismatched DP-record digest, altered commitment root, public-input disagreement, or receipt/artifact substitution.

#### 3.3.6. Evidence Guarantees and Boundaries

The evidence workflow supports identity-bound participation, explicit admission and inclusion outcomes, tamper-evident DP-record binding, canonical artifact integrity, deterministic model–anchor consistency reconstruction, replayable RSU model transitions, scoped Groth16 anchor-aggregation verification, and public receipt publication. These guarantees remain bounded rather than training-semantic: FL-BC-IDS verifies consistency among retained model artifacts and proof-oriented evidence under the declared deterministic contracts without claiming honest local training, benign updates, raw-data provenance, universal IoV security, or full semantic correctness of local learning.

### 3.4. Prototype Implementation and Runtime Workflow

**Figure 1:**
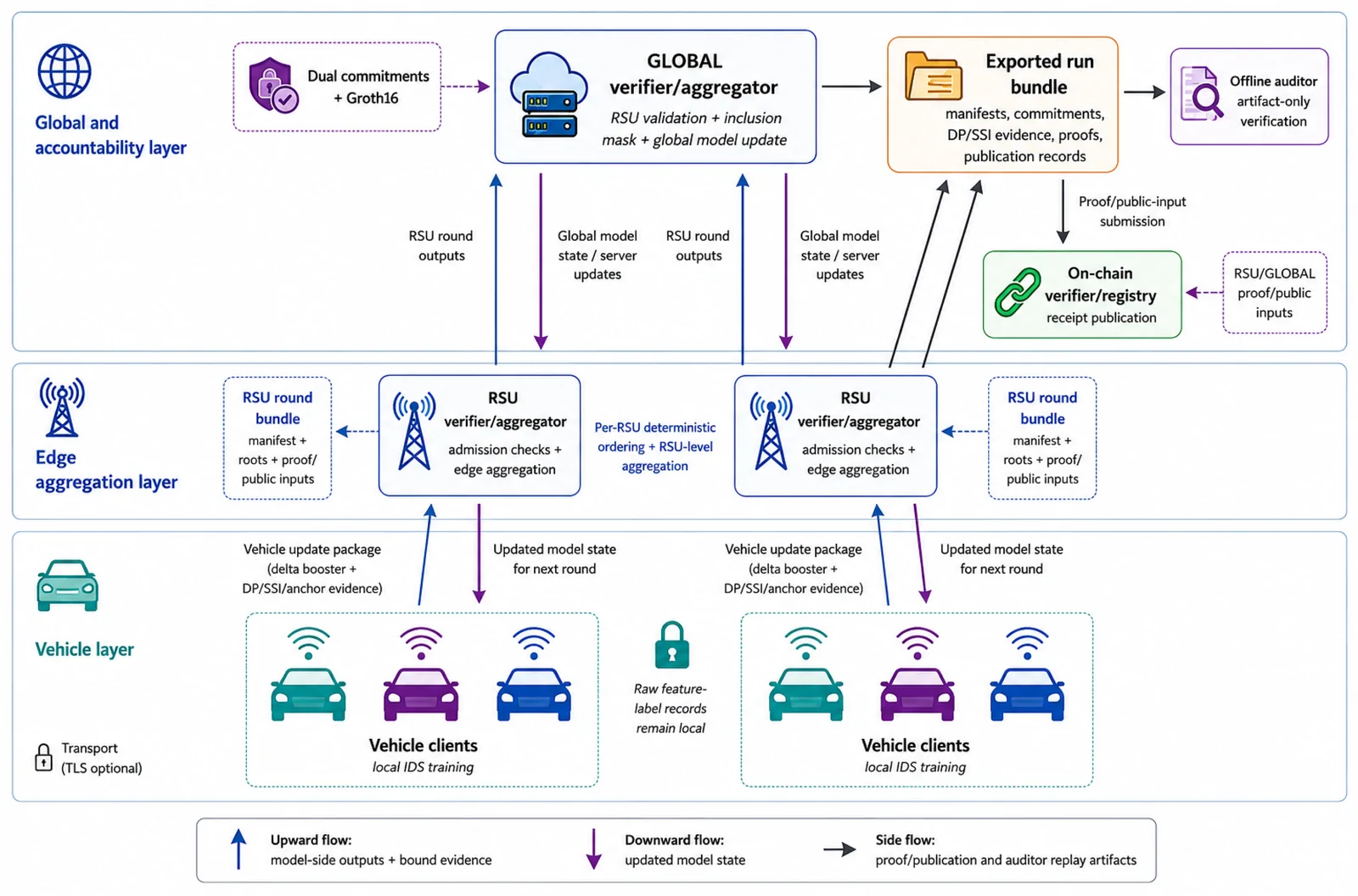
Layered prototype architecture of the implemented vehicle–RSU–GLOBAL federated IDS with supporting evidence, verification, and receipt-publication layers.

FL-BC-IDS was implemented as an executable software prototype following the vehicle–RSU–GLOBAL hierarchy defined in the proposed framework. The prototype was executed using CPython 3.12.6 on Windows 11 (10.0.26200) with a 64-bit x86 architecture (AMD64). Federated orchestration used flwr 1.23.0 and Ray 2.31.0. Learning and preprocessing used XGBoost 3.1.2, Sarus DP-XGBoost (dp_xgboost) 0.2.10, NumPy 1.26.4, pandas 2.3.3, SciPy 1.16.3, scikit-learn 1.7.2, Dask 2025.11.0, and imbalanced-learn 0.14.0. Figure 1 summarizes the implemented architecture and the relationship among the vehicle, RSU, GLOBAL, evidence-verification, and receipt-publication layers.

Groth16 proof generation and receipt-publication preparation used Node.js v24.0.2, Node Package Manager (npm) 11.3.0, Circom 0.5.46, snarkjs 0.7.5, and the universal phase-1 trusted-setup artifact powersOfTau28_hez_final_20.ptau before circuit-specific .zkey generation. The on-chain publication path used Sepolia verifier/registry contracts and constructed Solidity calldata (a,b,c,input) only after digest, public-input-length, and Barreto–Naehrig 254-bit pairing-friendly curve (BN254) field-bound checks passed [33,34].

Before federated execution, preprocessing produces the fitted transformer, feature contract, feature-domain bounds, and preprocessing manifest shared by the training, evidence-generation, and verification workflows. Dataset-specific preprocessing procedures are described in Section 3.5.1. Before DP-XGBoost training, transformed feature values are clipped according to Equation (13) using the run-level feature bounds exported by the preprocessing manifest. This clipping defines the bounded feature domain used by the DP learner; it is not a Byzantine-robust aggregation mechanism.

During federated execution within each RSU, a participating vehicle receives the current RSU booster when available, continues local DP-XGBoost training on its assigned training shard, and exports only the newly added serialized booster contribution rather than raw feature–label records.

The contribution is hashed and bound into a canonical signed vehicle participation report together with the DP-record digest, anchor digest, round identifier, RSU identifier, vehicle identifier, participant-identifier context, public key, and Ed25519 signature.

The prototype treats vehicle submissions and RSU bundles as explicit trust-boundary objects. Vehicle contributions are admitted only after the model-update digest, DP-record digest, anchor digest, Ed25519 signature, participant identifier, RSU scope, and federated-round context have been validated. Evidence-valid candidates are first ordered by (vehicle_id,did,cid) for deterministic capacity selection. After the admitted set is fixed, the corresponding Flower results are sorted by cid for XGBoost tree aggregation, and the same ordered set is recorded in the RSU manifest for canonical envelope and Merkle-root construction. Invalid contributions are excluded, with their exclusion reasons retained in the exported evidence.

At the GLOBAL scope, terminal RSU bundles are processed in ascending RSU-identifier order and are included only when their manifests, commitment roots, proof artifacts, public-input sidecars, and inclusion context validate successfully. The GLOBAL manifest records the aligned RSU identifiers, inclusion mask, RSU roots, and public binding values. The saved models of the included RSUs are evaluated under the common validation/test feature contract, and their bounded prediction vectors are combined using the equal-weight coordinate-wise clipped mean defined in Equation (26). If no vehicle is admitted at an RSU or no RSU satisfies the GLOBAL inclusion conditions, the corresponding verification or ensemble scope is marked invalid rather than producing a successful result.

Each completed run writes its configuration, preprocessing manifest, vehicle evidence, RSU manifests, GLOBAL manifest, proof/public-input artifacts, model snapshots, and publication records under one export directory. The offline verification workflow resolves a target scope from these retained artifacts, recomputes SHA-256 bindings, verifies Ed25519 signatures, checks deterministic model–anchor consistency, replays the recorded RSU model transition, reconstructs SHA-256 and Poseidon roots and expected public inputs, verifies the Groth16 proof, and cross-checks receipt pins when publication artifacts are present. A passing verdict establishes the declared bounded model/evidence consistency relations; it does not establish honest local training, raw-data provenance, correct runtime DP execution, benign updates, or full semantic correctness of the IDS.

### 3.5. Experimental Setup

This subsection defines the datasets, execution configurations, reference baselines, evidence checks, and evaluation metrics used to assess the implemented FL-BC-IDS prototype. The evaluation is designed to measure IDS utility, privacy–utility cost, evidence verification, receipt-publication behavior, runtime overhead, longer-round operation, controlled federated stress under non-IID data heterogeneity and unstable contribution availability, and expanded-topology evidence-workflow scaling under a controlled execution contract.

#### 3.5.1. Datasets and Preprocessing

The evaluation uses CSE-CIC-IDS2018 and CICIoV2024 to cover complementary IoV attack surfaces. CSE-CIC-IDS2018 represents external network-flow attacks, while CICIoV2024 represents internal CAN-bus attacks [11,12]. Both datasets are mapped to a binary classification task, BENIGN versus ATTACK. The use of the CAN-bus attack surface is additionally consistent with prior in-vehicle intrusion-detection research [15].

CSE-CIC-IDS2018 is treated as a tabular network-flow dataset, while CICIoV2024 is consumed in the decimal CAN-bus representation using the CAN arbitration identifier and data-byte fields under the selected feature contract [11,12,39]. Large raw inputs are loaded through Dask; labels are mapped into the binary BENIGN/ATTACK convention; numerical columns use median imputation and standardization; categorical columns, when present, use most frequent imputation and one-hot encoding; and the fitted transformer is learned from the training split only [40,41]. Train-only oversampling is applied to the training split before hierarchical vehicle partitioning when both classes are present and the configured size cap permits it; validation and test splits are never oversampled [42].

For the representative compact full-system execution, the main benchmark data are partitioned using deterministic stratified row-level 70/15/15 train/validation/test splits with random seed 42. The multi-seed statistical validation repeats the same partitioning procedure using seeds 42–51. Imputation, scaling, encoding, and the fitted transformation contract are learned from the training partition only and are then applied unchanged to the validation and test splits. This training-only fitting prevents validation/test leakage but does not make the fitted preprocessing quantities differentially private. Imbalance correction is restricted to training, and only the resulting training artifact is subsequently partitioned among RSUs and simulated vehicle clients. Validation and test data are never oversampled.

Table 3 summarizes the resulting preprocessed evaluation artifacts, feature counts, and evaluated attack surfaces. The training counts are reported after train-only imbalance correction, whereas validation and test retain their pre-correction post-split cardinalities.

The larger training counts in Table 3 therefore represent the post-correction training artifacts, whereas validation and test retain their pre-correction post-split cardinalities. The preprocessing implementations also retain source or input digests, feature and split metadata, fitted-transformer artifacts, and consistency checks on the exported data. These training-only preprocessing controls are distinct from cross-partition predictor recurrence, which is audited separately below.

For CICIoV2024, the six-decimal CAN source files are validated before merging for the expected file set, schema, labels, metadata, CAN identifier and data-byte domains, missing values, and agreement between the source-file class and the declared label. A separate raw-representation audit quantifies full-row repetition, exact ID+DATA_0,…,DATA_7 predictor repetition, consecutive identical-payload runs, and pairwise cross-file predictor overlap. Recurrent CAN payloads are retained because repetition is a property of the evaluated CAN traffic representation rather than being assumed a priori to constitute erroneous duplicated observations.

Table 4 summarizes the separation properties of the main benchmark splits and the generalization-validation protocols applied to each dataset.

As Table 4 shows, the main row-level benchmark splits are not claimed to provide chronological, source-vehicle, or exact predictor-pattern separation. Pattern-disjoint, grouping-based, supervised rolling-origin temporal, strict unseen-attack, and multiclass protocols provide complementary generalization tests under stronger separation conditions.

For the CSE-CIC-IDS2018 temporal evaluation, the deterministic source-wide model-side sample is sorted by timestamp and divided into ten approximately equal-size contiguous chronological intervals without splitting observations that share an identical timestamp. Nine consecutive transitions are evaluated. For each transition, all observations preceding the immediately prior interval together with the first 70% of that prior interval form the available training history. The final 30% of the prior interval is reserved exclusively for operating-point calibration, and the complete subsequent interval remains untouched until testing. The temporal-refresh evaluation is therefore a supervised periodic-update scenario: labels for the adaptation portion of the preceding interval are assumed to be available before evaluation of the subsequent interval.

Model preprocessing and the fixed 100-round XGBoost schedule are fitted using the available training history only. The primary operating threshold is then fixed exclusively from benign calibration scores using a 1% empirical false-positive-rate criterion. The temporal-refresh protocol, 70/30 adaptation–calibration split, transition set, model schedule, and threshold criterion were fixed before adaptive outcome evaluation; no labels or scores from the subsequent test interval participate in model fitting, operating-point selection, or endpoint definition. Temporal recall is evaluated only for test attacks whose attack type is already represented in the corresponding updated training history. Test attacks whose type is absent from that history are outside the seen-attack temporal endpoint and are treated as attack-novelty conditions.

For each strict leave-one-attack-out evaluation, the held-out attack is absent from both training and development data, predictor-hash disjointness is enforced across the evaluated partitions, and the operating threshold is likewise fixed from benign development scores using the fixed 1% false-positive-rate criterion. Their corresponding analyses and results are reported in Section 4.2. Only the training split is partitioned across the hierarchical FL topology. Within each seed-specific execution, the validation and test data remain fixed global evaluation artifacts. Training examples are distributed across RSU-local pools and vehicle shards using deterministic rules. The compact main runs retain balanced training shards and stable participation, whereas a separate controlled CSE-CIC-IDS2018 stress experiment introduces joint quantity, label, and feature heterogeneity together with scheduled persistent, isolated, and burst contribution unavailability. Mobility and seamless-handoff claims are not inferred from synthetic client assignments or from this FL-level availability stress.

#### 3.5.2. Execution Configurations and Evaluation Metrics

Table 5 summarizes the principal execution configurations used to evaluate IDS utility, DP-learner utility cost, evidence verification, runtime overhead, longer-round operation, controlled non-IID heterogeneity and participation stress, and expanded-topology evidence-workflow scaling. The compact full-system runs used four vehicles, two RSUs, two vehicles per RSU, two federated rounds per RSU, 10 local boosting iterations per vehicle per federated round, and num_parallel_tree = 1.

A separate controlled FL-level stress experiment was performed on CSE-CIC-IDS2018 using seed 42, two RSUs, 10 vehicles per RSU (20 vehicles in total), eight federated rounds, and 10 local boosting iterations per participating vehicle per round. The training artifact was repartitioned with joint local-data-volume and label heterogeneity with controlled feature-distribution skew. Client-volume weights were generated deterministically from a log-normal distribution with σ=0.90 and bounded by a requested maximum 10:1 size ratio. Client attack-propensity targets were drawn from a Beta(0.30,0.30) distribution and clipped to [0.01,0.99] before bounded integer allocation that preserved the exact global attack and benign totals. Feature heterogeneity was introduced by ordering a deterministically selected high-variance numeric feature within each class and allocating contiguous value bands across clients. Every training row was assigned to exactly one client, the original global class totals were preserved, feature values themselves were not modified, and the validation and test artifacts were left unchanged.

Participation stress was applied deterministically at the federated-round level. Within each RSU, one vehicle was unavailable during rounds 2–3, one had an isolated missed contribution in round 4, one became persistently unavailable from round 5 onward, and another was unavailable during rounds 6–7. Round 6 additionally exercised two signed-context admission challenges across the hierarchy: one vehicle contribution carried a stale round context, and one contribution was signed for a different RSU scope. Missing contributions were treated as unavailable rather than invalid, whereas received contributions with an incorrect signed context were subjected to the normal RSU admission checks. Admission and context verification remained active in every round. Full RSU commitment/proof artifacts were materialized for the challenge round and final round, and the final GLOBAL proof was generated from the terminal validated RSU scopes. This selective materialization changed only the frequency of complete proof-artifact generation in this stress evaluation; it did not change local training, admission, context verification, or RSU aggregation.

Under the same conditional pure-ϵ learner-stage accounting used for the compact experiments, the per-round bound remains $\epsilon_{\mathrm{round}}\leq 0.5525028434$. Therefore, for a vehicle that participates in all eight federated rounds, the applicable learner-stage analytical bound is

(31) $$\epsilon_{\mathrm{total},8}\leq 8\times 0.5525028434=4.4200227469\approx 4.4200.$$

Scheduled unavailability cannot increase this learner-stage bound. The calculation uses δ=0, basic composition, and no privacy credit for early stopping. As in the compact evaluation, it applies to learner-input record instances conditional on the fitted preprocessing artifact and is not an end-to-end original-record privacy budget.

For multi-seed statistical validation, the centralized, matched non-DP FL, and FL-BC-IDS learning configurations were additionally evaluated across 10 seeds (42–51). Each seed generated a distinct data partition shared by the three configurations for paired comparison. Results are summarized by the mean, standard deviation, and 95% bootstrap confidence interval. Statistical significance was assessed using two-sided exact paired permutation tests with Holm correction for multiple comparisons.

In both datasets, the recorded configuration used dp_enabled = true, tree_method = approxDP, $\epsilon_{\mathrm{tree}}=0.25$, subsample = 0.2, num_parallel_tree=1, and δ=0. Using the conditional learner-stage record accounting, subsampling amplification, and basic composition defined in Section 3.2.5, the resulting bounds are those derived in Equations (18) and (19): $\epsilon_{\mathrm{round}}\leq 0.5525$ and $\epsilon_{\mathrm{total}}\leq 1.1050$, respectively, for one learner-input record instance over the two-round compact configuration. These values are distinct from the original-record account in Equations (20) and (21).

Both compact main runs used balanced training partitions, with scale_pos_weight = 1.00.

Matched non-DP hierarchical references preserved the same train/validation/test splits, balanced two-RSU/four-vehicle topology, two federated rounds per RSU, 10 local boosting iterations per vehicle per federated round, num_parallel_tree = 1, RSU-wise evaluation, and GLOBAL ensemble evaluation.

The ablation differed only in that the differential-privacy mechanism and its associated accounting were disabled.

Centralized non-FL XGBoost baselines used the same preprocessed train/validation/test splits and binary label mapping, but removed FL partitioning, RSU/GLOBAL aggregation, DP, proof generation, and receipt publication.

The expanded-topology evidence-workflow scaling runs used 10 RSUs and 20 vehicles per RSU, yielding 200 vehicles in total. These runs preserved two federated rounds per RSU, 10 local boosting iterations per vehicle per federated round, num_parallel_tree = 1, tree_method = approxDP, and the same recorded privacy-budget family. The proof capacities were enlarged to $K_{\max}=20$ for RSU-scope vehicle capacity, $R_{\max}=10$ for GLOBAL-scope RSU capacity, $N_{\max}=20$ for GLOBAL-scope vehicles-per-RSU capacity, and M=64 for the anchor-vector length.

The 20-round CSE-CIC-IDS2018 run used the compact two-RSU/four-vehicle topology to characterize longer-horizon RSU-level validation behavior beyond the two-round benchmark configuration.

The evaluation reports eight evidence dimensions: DP-record integrity, participation verification, deterministic model–anchor consistency, recorded model-transition replay, deterministic commitment reconstruction, Groth16 proof and public-input agreement, on-chain receipt consistency, and minimal on-chain disclosure. These dimensions are interpreted together with IDS utility, DP-learner utility comparisons, runtime overhead, publication cost, longer-round operation, controlled non-IID heterogeneity and participation stress, and expanded-topology evidence-workflow scaling.

The reported predictive metrics include accuracy, precision, recall, F1-score, area under the receiver operating characteristic curve (ROC-AUC), log-loss, Brier score, expected calibration error (ECE), true negatives (TN), false positives (FP), false negatives (FN), and true positives (TP). System-level measurements include runtime, communication volume, gas, Ether (ETH) cost, and proof/publication counts.

The Sepolia publication evaluation records proof-transaction inclusion latency, transaction-total latency, consensus-finality latency, observed transaction and proof-verification failures, finality timeouts, and the exercised transaction-submission pattern. Transaction inclusion and consensus finality are evaluated as distinct publication stages to characterize both receipt availability and finalized on-chain confirmation. Latency measurements are summarized using the mean, median, and 95th percentile (p95).

Confusion matrices follow the standard binary form

(32) $$\left[\begin{array}{cc} TN & FP\\ FN & TP \end{array}\right].$$

## 4. Results and Discussion

This section reports and discusses IDS utility, generalization stress tests, 20-round behavior, controlled federated heterogeneity and participation stress, privacy–utility and baseline comparisons, verification and publication outcomes, runtime overhead, and expanded-topology evidence-workflow scaling.

### 4.1. End-to-End IDS Performance

Table 6 reports the final GLOBAL ensemble results for the two representative seed-42 compact full-system runs. The controlled principal benchmark results show high predictive utility on both datasets under the privacy-aware evidence workflow. CICIoV2024 exhibited fewer classification errors at the selected decision threshold, whereas CSE-CIC-IDS2018 also achieved high principal-benchmark performance but produced a larger number of false negatives. These results characterize the controlled main benchmark splits; distribution-shift and unseen-attack behavior are evaluated separately in Section 4.2.

Using the entry ordering defined in Equation (32), the CSE-CIC-IDS2018 GLOBAL ensemble confusion matrix is

(33) $$\left[\begin{array}{cc} 422427 & 216\\ 622 & 26736 \end{array}\right].$$

For CICIoV2024, the GLOBAL ensemble confusion matrix is

(34) $$\left[\begin{array}{cc} 183504 & 57\\ 4 & 27668 \end{array}\right].$$

**Figure 2:**
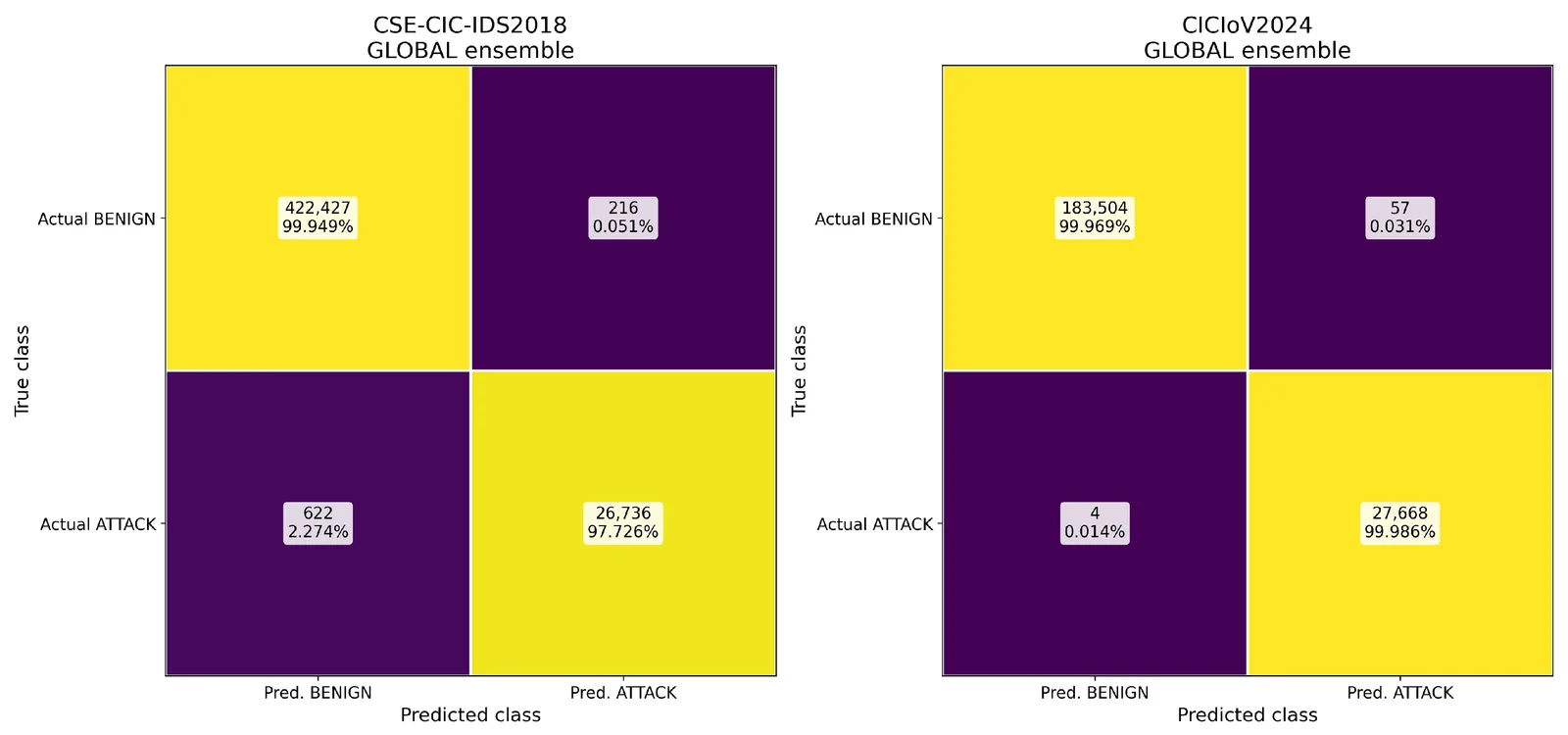
Cross-dataset comparison of the full-system GLOBAL ensemble confusion matrices.

These counts show that both datasets reached high overall performance, but CICIoV2024 produced a much lower error mass under the compact full-system configuration. Figure 2 visualizes the cross-dataset confusion-matrix comparison.

The cross-dataset results reveal distinct error profiles. CSE-CIC-IDS2018 uses a higher-dimensional flow-level feature representation and a larger test set; under the compact DP-enabled hierarchical setting, it produced a higher false-negative count. CICIoV2024, by contrast, remained close to perfect thresholded classification, with only four false negatives. This does not mean that CICIoV2024 is inherently easier in all IoV settings; it means that, under the selected binary CAN-bus representation and controlled split used here, the implemented workflow produced a cleaner operating point.

### 4.2. Dataset Validation and Generalization Stress Tests

The main FL-BC-IDS experiments evaluate the complete hierarchical privacy, evidence, proof, and publication workflow under the fixed stratified benchmark artifacts described above. Dataset generalization is evaluated separately at the classifier level using the same dataset representations and XGBoost model family under progressively stronger separation conditions. This isolates the influence of split structure and attack-distribution shift from the end-to-end FL, DP, evidence, and Groth16 workflow.

A hash-based audit of the exact saved main artifacts, with labels excluded from the predictor hash, found limited cross-split exact-vector recurrence in CSE-CIC-IDS2018: 1376 of 450001 test rows (0.306%) have an exact predictor vector occurring in training. CICIoV2024 is structurally much more repetitive under the selected decimal CAN representation: 210792 of 211233 test rows (99.791%) have an exact predictor vector represented in training. Across the complete 1,408,219-row decimal representation, only 3588 distinct ID+DATA_0,…,DATA_7 predictor vectors occur, and the audit found no predictor hash shared by different classes. This finding demonstrates extensive cross-partition predictor recurrence in the selected CAN representation. It is analytically distinct from leakage caused by fitting preprocessing operations on validation or test data: the preprocessing pipeline is training-fitted, whereas the main row-level benchmark partitioning can still place recurrent predictor patterns in more than one split. The pattern-disjoint and unique-pattern evaluations below address this second form of dependence directly.

The strongest duplicate-control result was obtained on CSE-CIC-IDS2018. When exact raw predictor hashes were treated as indivisible groups, producing approximately 70/15/15 partitions with zero train–validation, train–test, or validation–test hash overlap, the binary classifier achieved 0.999718 test accuracy, 0.997744 recall, 0.997690 F1-score, and 0.999959 ROC-AUC. Collapsing repeated test rows to one equal-weight observation per distinct unambiguous predictor pattern yielded almost identical accuracy (0.999717) and F1-score (0.997668). A separate Flow-ID-disjoint test achieved 0.999705 accuracy, 0.997529 recall, 0.997583 F1-score, and 0.999973 ROC-AUC. Flow ID is interpreted only as the grouping identifier supplied by the dataset and not as proof of higher-level application session separation.

Temporal generalization on CSE-CIC-IDS2018 was evaluated using the supervised rolling-origin protocol defined in Section 3.5.1. The timestamp-ordered sample was divided into ten contiguous intervals without splitting identical timestamps, yielding nine consecutive chronological transitions. In each transition, earlier observations and the first 70% of the immediately preceding interval formed the available training history, the final 30% was used exclusively for operating-point calibration, and the complete subsequent interval remained untouched until testing. The primary decision threshold was fixed solely from benign calibration scores using the fixed 1% empirical false-positive-rate criterion.

Across the eligible chronological transitions, 6968 test attack observations belonged to attack types already represented in the updated training history. The pooled seen-attack recall was 0.984788 at a pooled test FPR of 0.005700. Moreover, 6320 of these observations (90.7%) had predictor hashes absent from the corresponding training history, and recall on this predictor-hash-novel subset remained 0.983228. This result shows that the high pooled temporal recall is not attributable to exact predictor-pattern recurrence. Chronological intervals without a previously represented attack type were outside the seen-attack temporal endpoint and were therefore not assigned a seen-attack temporal recall.

Table 7 summarizes the principal held-out results of the generalization stress tests.

Unseen-attack behavior was evaluated independently using a strict leave-one-attack-out protocol. For each experiment, the target attack family or attack type was completely absent from model fitting and development/calibration data. Benign and remaining seen-attack records were partitioned by predictor hash, and zero predictor-hash overlap was enforced across the evaluated partitions. The operating threshold was fixed exclusively from benign development scores using the fixed 1% empirical false-positive-rate criterion; no held-out attack sample participated in model fitting or threshold selection. Table 8 summarizes the resulting dataset-level unseen-attack performance.

The strict unseen-attack evaluation produced a macro-averaged recall of 0.8031 across seven held-out CSE-CIC-IDS2018 attacks and 0.9090 across five held-out CICIoV2024 attack families, with median recalls of 1.0000 and 0.999946, respectively. These results quantify generalization to attack types excluded completely from training and calibration under a leakage-free, development-calibrated evaluation contract.

Multiclass recognition provides a further check that the selected representations are not evaluated only through an aggregated BENIGN/ATTACK label. The strict predictor-hash-group-disjoint CSE-CIC-IDS2018 experiment retained nine classes and achieved 0.991180 accuracy, 0.994269 balanced accuracy, and 0.943428 macro-F1. The equal-weight unique-pattern evaluation remained nearly identical, at 0.991159 accuracy and 0.943475 macro-F1.

For CICIoV2024, limited distinct predictor-pattern support in several spoofing classes prevents construction of a three-way split that simultaneously preserves all six classes in training, validation, and test while maintaining strict predictor-hash disjointness. Accordingly, the multiclass evaluation uses a strict two-way train/test predictor-hash-group-disjoint protocol with all six classes represented in both partitions, a predefined XGBoost configuration, a fixed 100-round training schedule, no validation-based early stopping, and no test-driven model selection. Train–test predictor-hash overlap is zero. Under this strict zero-overlap protocol, the classifier achieved 0.999779 accuracy, 0.999955 balanced accuracy, and 0.999246 macro-F1. These results characterize multiclass recognition under complete exact predictor-pattern separation between training and testing.

Finally, source-vehicle-disjoint evaluation is not claimed. The simulated FL vehicle identifiers identify deterministic training shards; they are not substituted for physical source-vehicle identities. CSE-CIC-IDS2018 does not provide a suitable source-vehicle identifier for this purpose, and the selected CICIoV2024 decimal representation contains neither source-vehicle, timestamp, nor session identifiers. The corresponding separation properties are therefore reported as unavailable rather than inferred from synthetic client assignments.

### 4.3. Twenty-Round Federated Learning Behavior

The compact full-system benchmarks use two federated rounds per RSU to exercise the complete learning, evidence, proof-generation, and receipt-publication workflow. A 20-round CSE-CIC-IDS2018 run was also executed to characterize RSU-level learning behavior over a longer training horizon.

The 20-round run used the same privacy-aware approxDP training family, two RSUs, two vehicles per RSU, 10 local boosting iterations per vehicle per federated round, and 20 federated rounds per RSU.

Under the same conditional pure-ϵ learner-stage accountant used for the compact experiments, the per-round bound derived in Equation (18) remains $\epsilon_{\mathrm{round}}\leq 0.5525$; therefore, 20 complete federated rounds correspond to the learner-stage analytical upper bound

(35) $$\epsilon_{\mathrm{total},20}\leq 20\times 0.5525028434=11.0500568674\approx 11.0501$$

for a learner-input record instance, without credit for possible early stopping. This value retains the same preprocessing and adjacency conditions as the compact account and is not an end-to-end original-record privacy budget. The 20-round experiment is consequently used to demonstrate execution depth and learning behavior, not to suggest that it retains the two-round learner-stage budget.

**Figure 3:**
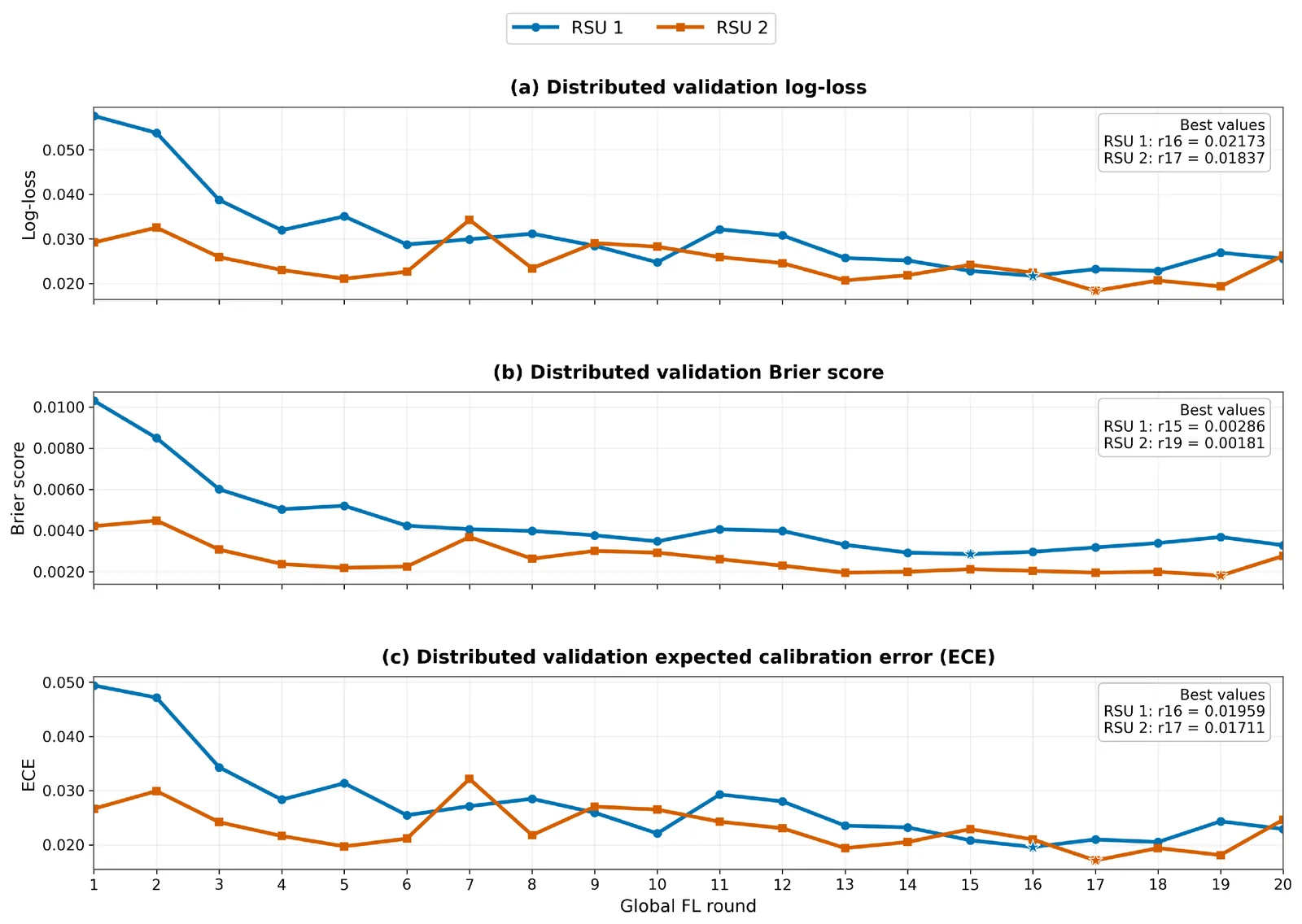
Twenty-round CSE-CIC-IDS2018 validation trajectories of the two RSU-level federated models. The top panel shows distributed validation log-loss, the middle panel shows distributed validation Brier score, and the bottom panel shows distributed validation expected calibration error (ECE). Lower values indicate lower validation error or calibration deviation, and the star markers identify the minimum observed value for each RSU trajectory.

The retained artifacts support RSU-level round-wise validation curves rather than a true round-wise GLOBAL ensemble curve because the RSU summary files store complete per-round histories, whereas the saved GLOBAL ensemble summary contains only the final ensemble outcome. For this reason, Figure 3 reports the distributed validation trajectories of the two RSU-level federated models rather than inferring an unsupported GLOBAL round-wise trajectory.

The 20-round trajectories show that the system continued to operate beyond the compact two-round benchmark and that both RSU-level models improved substantially during the earlier federated rounds. Because lower values are preferable for log-loss, Brier score, and ECE, the initial downward trajectories indicate reduced validation error and calibration deviation. The minimum log-loss was 0.02173 at round 16 for RSU 1 and 0.01837 at round 17 for RSU 2. The minimum Brier scores were 0.00286 at round 15 and 0.00181 at round 19, respectively, while the minimum ECE values were 0.01959 at round 16 and 0.01711 at round 17. The later trajectories remain in a comparatively low-error regime but exhibit non-monotonic fluctuations, showing that additional rounds do not guarantee continued improvement and that the final round is not necessarily the validation-optimal checkpoint.

The DP-XGBoost configuration, privacy budget per tree, subsampling fraction, number of local boosting iterations, topology, and other training parameters were held fixed throughout the 20-round run. Accordingly, the above figure characterizes the dependence of the validation measures on federated-round progression under this fixed operating configuration; it does not isolate the causal influence of individual hyperparameters. Assessing such effects would require a dedicated parameter-ablation study. The RSU-to-RSU differences should likewise be interpreted as distinct validation trajectories under their respective training shards rather than as evidence that one RSU configuration is intrinsically superior.

### 4.4. Controlled Non-IID Heterogeneity and Participation Stress

A separate controlled FL-level stress experiment was performed on CSE-CIC-IDS2018 to evaluate non-IID quantity, label, and feature heterogeneity together with unstable participation, independently of the topology-scaling experiment. The combined stress condition used 20 vehicles, two RSUs, eight federated rounds, and 10 local boosting iterations per participating vehicle per round, while retaining the same unchanged validation and test artifacts.

The heterogeneous partition produced client training sets ranging from 60,353 to 433,518 samples, corresponding to a 7.18× maximum-to-minimum size ratio, while realized local attack fractions ranged from 0.00879 to 0.87028. Feature-distribution skew was introduced through contiguous within-class value bands of the deterministically selected high-variance training feature, without modifying the feature values themselves. Every training row remained assigned exactly once and the global attack/benign totals were preserved. Table 9 summarizes the evaluated stress dimensions and the corresponding observed outcomes.

Across the 160 scheduled vehicle-round fit opportunities, 142 completed and 18 were intentionally unavailable, yielding an observed participation rate of 0.8875. In the challenged sixth round, each RSU received eight successful fit results. The stale-round contribution at RSU 1 and the cross-RSU-scope contribution at RSU 2 were rejected by admission, leaving seven admitted contributions at each RSU while aggregation continued with the remaining valid clients. At the terminal eighth round, nine contributions were admitted at each RSU.

Under the joint non-IID condition—comprising quantity heterogeneity, label-distribution skew, and feature-distribution skew— together with unstable participation, the final GLOBAL ensemble achieved 0.998151 accuracy, 0.985575 precision, 0.983990 recall, 0.984782 F1-score, and 0.998544 ROC-AUC on the unchanged test set. These values therefore represent the observed learning outcome of the combined non-IID and participation-stress experiment, rather than an IID or balanced-shard evaluation. The materialized RSU anchor-aggregation proof scopes for the challenge and terminal rounds were verified successfully, as did the final GLOBAL proof. At the configured terminal audit scope, independent commitment recomputation and authoritative public-input reconstruction also passed.

This experiment extends the evaluation beyond balanced shards and stable full participation, but its interpretation remains at the controlled FL-orchestration level. Scheduled unavailability represents round-level contribution availability rather than packet-level wireless link behavior; the stale-round challenge evaluates admission of a delayed or stale signed context rather than arbitrary asynchronous optimization; and the cross-RSU challenge verifies RSU-scope binding rather than seamless handoff continuity. Physical mobility and stateful handoff therefore remain separate deployment questions.

### 4.5. DP-Learner Utility and Baseline Comparisons

Table 10 reports two controlled reference configurations designed to isolate specific design choices of FL-BC-IDS rather than to construct a cross-paper performance leaderboard. The matched non-DP hierarchical reference preserves the same FL hierarchy, data splits, model family, rounds, and evaluation workflow while disabling the DP-aware learner mode and its associated privacy accounting, thereby isolating the utility effect of the DP-enabled learner under the shared preprocessing contract. The centralized XGBoost reference uses the same preprocessed train/validation/test artifacts and model family while removing FL partitioning, raw-data locality, RSU/GLOBAL aggregation, DP, proof generation, and publication, thereby providing an unconstrained predictive reference.

Direct numerical reproduction of heterogeneous external federated IDS, Byzantine-robust FL, blockchain-assisted FL, and verifiable-computation systems is not treated as an equivalent controlled baseline because these approaches differ in their learning model, aggregation semantics, privacy mechanism or absence thereof, threat objective, verification statement, dataset representation, and execution architecture. Imposing identical privacy budgets, model capacity, aggregation rounds, and proof semantics would therefore require method-specific redesign rather than faithful reproduction. Their security and evidence capabilities are instead compared explicitly in Section 2 and Table 1.

**Figure 4:**
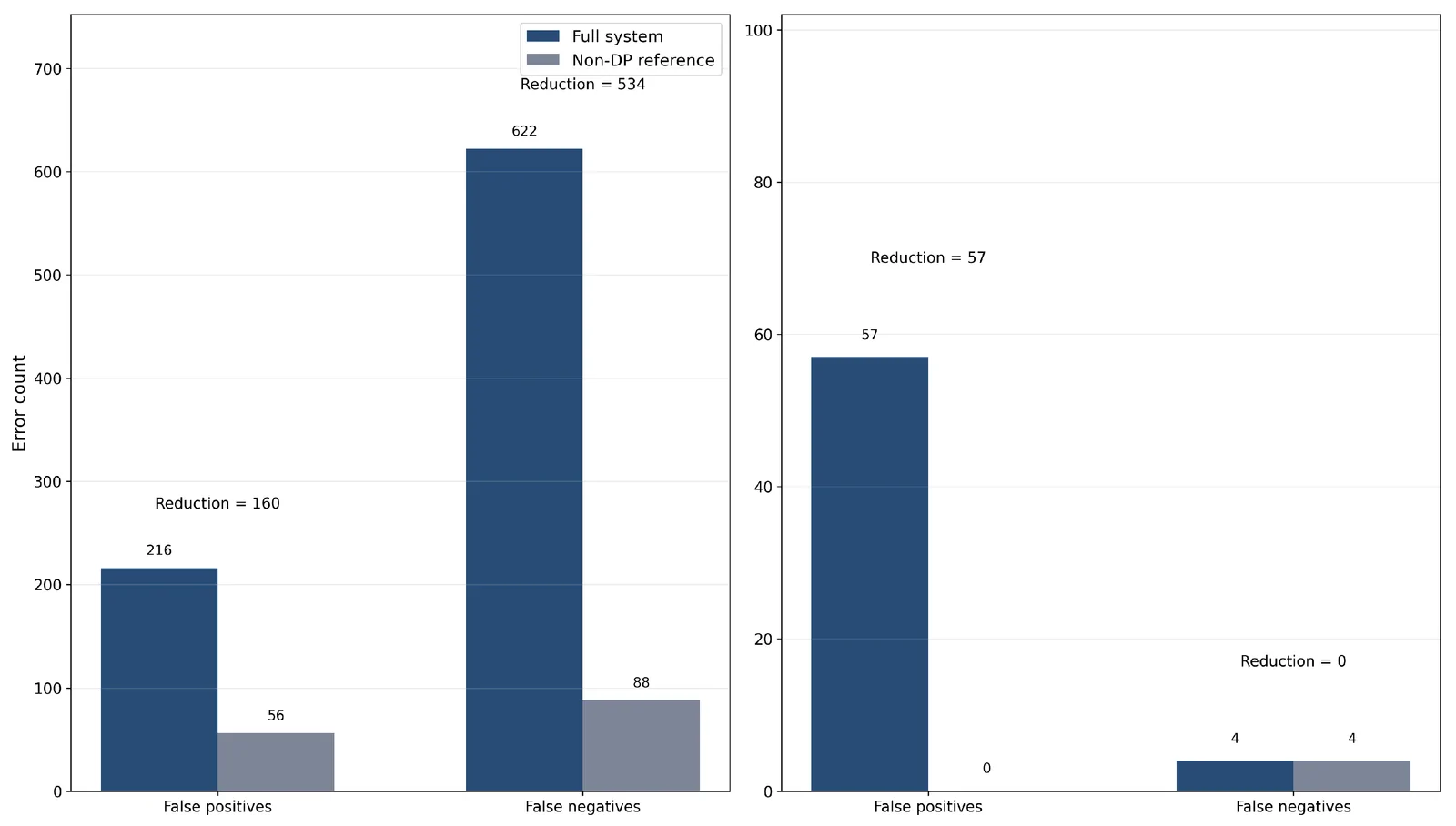
Error count comparison between the DP-enabled full system and matched non-DP hierarchical references.

On CSE-CIC-IDS2018, the non-DP reference reduced false positives from 216 to 56 and false negatives from 622 to 88. The centralized baseline reduced false positives to 18 and false negatives to 141. On CICIoV2024, the non-DP reference eliminated all 57 false positives while keeping the false-negative count unchanged at four, and the centralized baseline further reduced false negatives from four to two. Figure 4 highlights the error count difference between the DP-enabled full system and the matched non-DP hierarchical references.

These comparisons should not be interpreted as a direct ranking of predictive performance. The full FL-BC-IDS retains the DP-enabled learner and complete evidence workflow under the common fitted preprocessing contract. The non-DP reference keeps the FL structure but removes the DP/privacy-evidence layer. The centralized baseline removes FL partitioning and raw-data locality by training on centralized data. Therefore, the higher scores of the references are expected because they operate with fewer constraints.

**Multi-seed statistical validation.** To assess robustness to seed and partition variation, the principal learning comparison was repeated across 10 seeds (42–51). Table 11 reports the resulting mean, standard deviation, and 95% bootstrap confidence interval.

The paired statistical analysis confirmed significant differences among the three learning configurations on CSE-CIC-IDS2018 after Holm correction ($p_{\mathrm{adj}}<0.05$ for all tested pairwise method–metric comparisons). For CICIoV2024, the Holm-corrected tests did not identify statistically significant differences among the near-ceiling results ($p_{\mathrm{adj}}>0.05$). Thus, comparative conclusions are based on multi-seed statistical evidence rather than isolated six-decimal-point estimates.

The main conclusion is that FL-BC-IDS is not designed only to maximize unconstrained classifier scores. Its contribution is to preserve high predictive utility on the controlled principal benchmarks while retaining raw-data locality, declared learner-stage DP-accounting evidence, accountable participation, deterministic commitments, proof/public-input agreement, and digest-pinned public receipt publication. The generalization analysis additionally reports high pooled seen-attack recall under supervised temporal refresh for previously represented attack types while evaluating completely unseen attack families under a separate strict held-out protocol.

### 4.6. Evidence Verification and Publication Outcomes

The retained artifacts support successful RSU-scope and GLOBAL-scope verification for the evaluated compact full-system runs. In the main benchmark runs, both RSUs were included, no client was excluded, no capacity truncation occurred, and the evaluated RSU/GLOBAL proof scopes completed successfully. Table 12 summarizes the proof-verification and receipt-publication outcomes.

Table 13 reports the controlled security-evidence checks used to test invalid signatures, DP-binding mismatches, tamper detection, commitment recomputation, and public-input agreement.

These checks show that invalid or inconsistent evidence is explicitly rejected or detected and that valid exported artifacts can be independently reconstructed at the declared proof scope. Corrupted Ed25519 signatures were rejected before aggregation, mismatched DP-record bindings excluded the participant, and copied or modified DP records were detected through digest mismatch. For valid exported evidence, the auditor recomputed the SHA-256/Poseidon commitment roots and reconstructed the expected Groth16 public-input vector, with exact agreement on both datasets. The interpretation remains bounded: the evaluation verifies artifact consistency, digest binding, commitment recomputation, public-input agreement, proof/publication consistency, and selected controlled failure behavior. These controlled checks evaluate artifact integrity, evidence binding, and reconstruction behavior; they are not a benchmark of Byzantine-robust or poisoning-robust collaborative learning. Accordingly, they do not establish resistance to poisoned or backdoored updates, colluding participants, Byzantine or malicious aggregators, Sybil enrollment, or compromised signing keys.

A separate controlled model-linkage and aggregation-replay validation was performed to evaluate the computational consistency relations targeted by the evidence verifier. The evaluated cases and outcomes are summarized in Table 14.

The controlled validation directly exercised the model–anchor mismatch counterexample. A canonical report containing the digest of one serialized XGBoost contribution and the anchor digest associated with a different contribution retained a valid Ed25519 signature, but the independent model–anchor consistency check rejected the pair as a derivation mismatch. Model-update substitution and anchor-vector substitution were likewise rejected. The deterministic replay tests also confirmed reproducibility under identical ordered model artifacts and detected ordering changes, aggregate modification, and incompatible model contracts. These checks are verification-layer tests and do not alter the predictive-learning results reported elsewhere in this section.

Table 15 reports the Sepolia publication outcomes, including proof verification, publication cost, transaction latency, consensus finality, observed failures, and submission behavior. The dataset-specific gas totals include deployment, proof submission, and run-finalization transactions, and therefore should not be interpreted as proof-submission gas alone. The latency and finality rows report a single instrumented five-proof publication execution and are therefore shown once across the two dataset columns.

In the instrumented Sepolia publication execution, transaction inclusion and consensus finality were measured separately. All five proof-publication transactions were mined successfully and produced successful Groth16 verification outcomes, with no observed transaction or proof-verification failure. The mean proof-transaction total latency was 13.685 s (median 11.758 s; p95 21.917 s), while the mean receipt/inclusion wait latency was 11.972 s (median 10.098 s; p95 20.163 s). All six monitored publication transactions, comprising the five proof transactions and the run-finalization transaction, reached consensus finality without timeout. Across the five proof transactions, the mean receipt-to-finality latency was 1093.514 s (18.23 min; median 1093.134 s; p95 1117.961 s), while the mean transaction-start-to-finality latency was 1107.199 s (18.45 min; median 1104.498 s; p95 1139.878 s).

**Figure 5:**
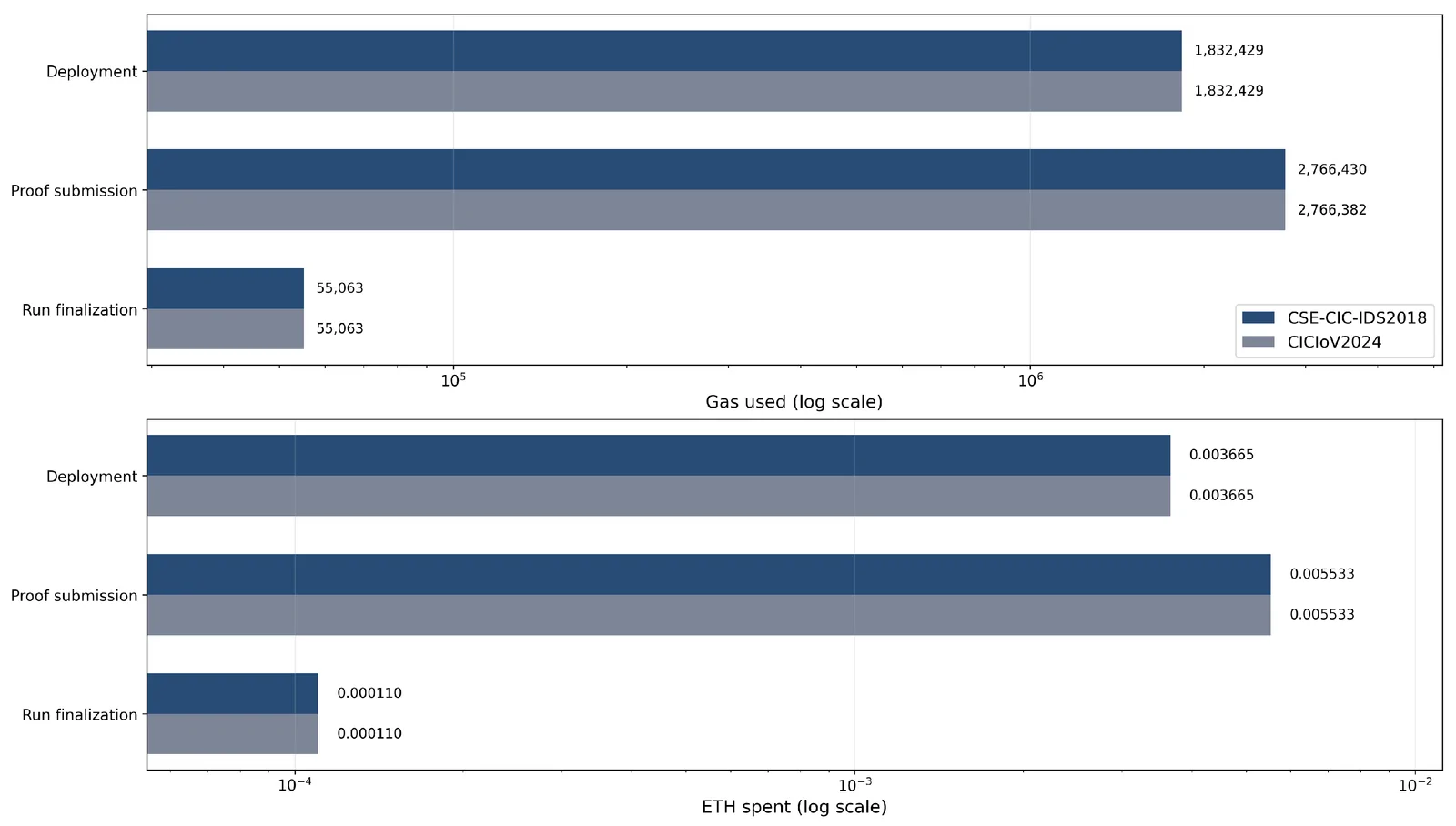
Stage-wise on-chain publication footprint for the compact Sepolia publication runs.

Figure 5 shows the stage-wise on-chain publication footprint for the compact Sepolia publication runs.

The publication footprint is nearly identical across the two datasets because the same number and type of proof submissions were published. This supports the intended narrow on-chain role: the blockchain stores accepted proof/public-input outcomes and fixed-length digest pins, not raw telemetry, model updates, or per-client evidence records.

The Sepolia evaluation exercised the complete publication path, including proof verification, transaction inclusion, consensus finality, storage read-back, duplicate-proof-key rejection, single-finalization enforcement, and GLOBAL-proof-gated run finalization. All monitored publication transactions reached finality without timeout, and no transaction or proof-verification failures were observed. The reported latency and finality measurements correspond to the evaluated serial single-sender publication pattern. Sepolia is used here as an Ethereum application-development test network with a permissioned validator set; accordingly, these measurements are not interpreted as evidence of production-mainnet decentralization or mainnet-level economic security. Broader concurrent and production deployment conditions remain outside the scope of this prototype evaluation.

### 4.7. Runtime and Layered Overhead

Table 16 summarizes the coarse runtime decomposition for the compact full-system runs. CSE-CIC-IDS2018 required substantially greater client wall time and local training time, primarily because it involved a much larger dataset and a higher-dimensional feature representation. The GLOBAL aggregation, proof, and export stage remained close to one second on both datasets.

**Figure 6:**
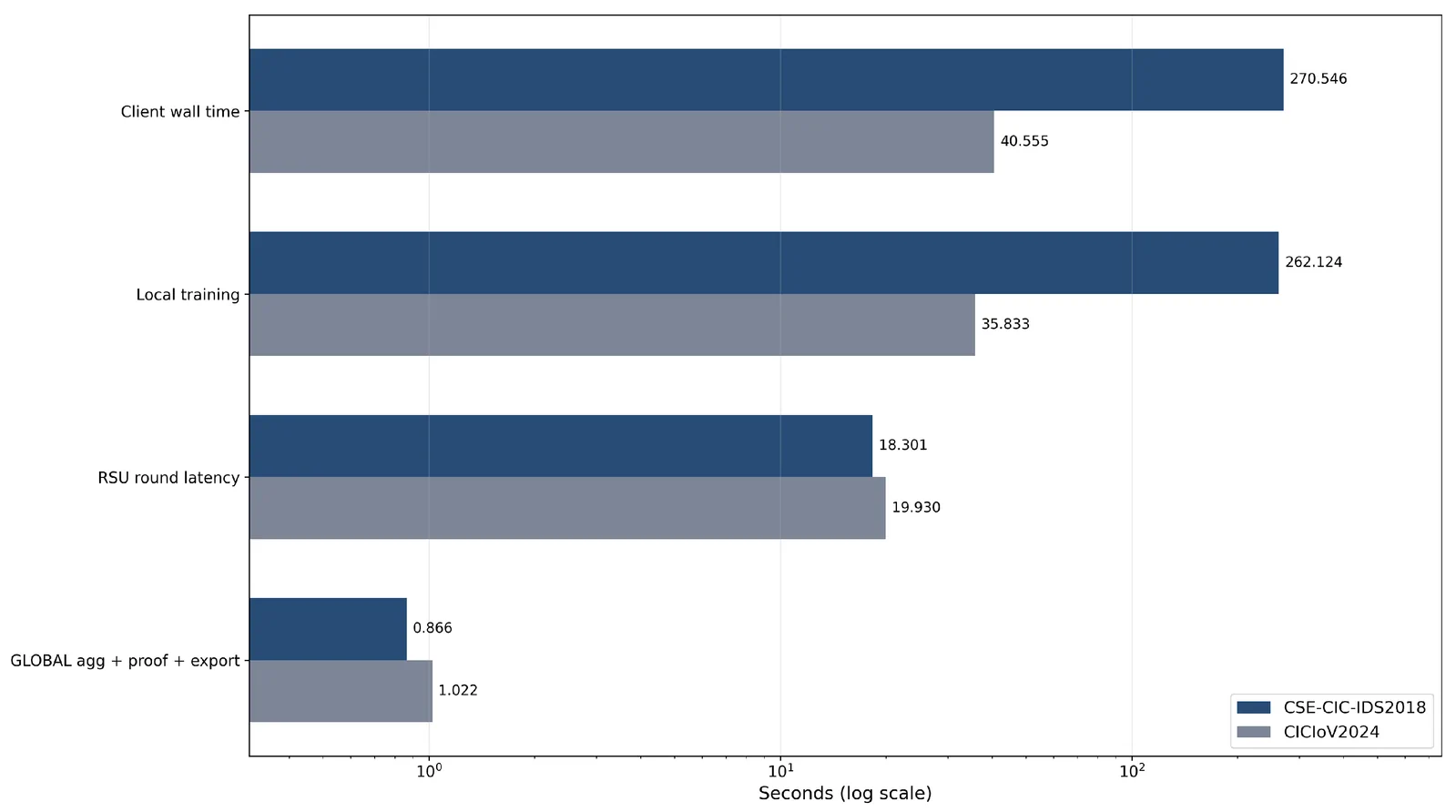
Cross-dataset runtime decomposition of the compact full-system runs on a log scale.

Figure 6 visualizes the same compact runtime decomposition on a log scale, making the relative difference between the learning and evidence-processing stages easier to compare.

The compact security-evidence ablations provide a clearer decomposition of off-chain evidence overhead. Table 17 shows that local learning dominated CSE-CIC-IDS2018, while CICIoV2024 had a smaller learning core and therefore made fixed evidence-processing costs more visible.

The overhead pattern is consistent across datasets. DP evidence generation was negligible in absolute time. Commitment construction and proof generation/verification were the largest non-learning costs. Therefore, future optimization should focus more on commitment/proof processing and publication batching than on DP-record export.

### 4.8. Expanded-Topology Evidence-Workflow Scaling

Table 18 compares the evidence-publication footprint of the compact two-RSU/four-vehicle hierarchy with the expanded 10-RSU/200-vehicle hierarchy. The purpose of this experiment is to characterize structural growth of the proof, export, and publication workflow as the number of participating scopes increases; it is not used as a deployment-validity or predictive-invariance test. The expanded configuration increased the number of submitted proof scopes from 5 to 21 per dataset while preserving the same fixed-format publication interface.

The expanded hierarchy therefore exercises a larger number of vehicle, RSU, proof, export, and publication scopes without changing the fixed-format receipt interface. This experiment is to be interpreted only as evidence-workflow scaling; predictive robustness under larger, heterogeneous, mobile, or intermittently connected deployments remains a separate evaluation question.

Publication cost scaled with the number of submitted proof scopes. The compact runs published 5 proofs, while each expanded run published 21 proofs. Gas increased from about 4.65 million to about 13.39 million, and the recorded ETH amount increased from about 0.0093 to about 0.0268 per dataset. This confirms that the publication interface remains fixed-format per scope, while total cost grows with the number of scopes submitted.

### 4.9. Overall Discussion

The results support the main claim of FL-BC-IDS under a bounded interpretation. The compact full-system runs achieved high predictive utility on the controlled principal benchmark splits while preserving raw-data locality and adding declared conditional learner-stage DP evidence, identity-bound participation, deterministic commitments, scoped Groth16 verification, public-input reconstruction, and digest-pinned blockchain receipts. The supervised rolling-origin temporal evaluation achieved a pooled seen-attack recall of 0.984788 at a pooled test FPR of 0.005700, with 0.983228 recall retained across 6320 predictor-hash-novel temporally shifted attack observations. The strict held-out-attack evaluation further produced a macro-averaged recall of 0.8031 on CSE-CIC-IDS2018 and 0.9090 on CICIoV2024 under development-only operating-point calibration. Together, these results separate temporal transfer of previously represented attack types from completely unseen attack families while preserving the central contribution of this article: a privacy-aware and independently checkable federated IDS workflow.

The multi-seed analysis provides statistically supported configuration differences on CSE-CIC-IDS2018, while all three configurations remained at near-ceiling predictive levels on CICIoV2024. Accordingly, the DP-learner utility comparison is grounded in multi-seed statistical evidence rather than isolated six-decimal-point estimates.

The separate model-linkage validation further showed that a correctly signed report pairing a model-update digest with an anchor derived from an unrelated model artifact failed the independent model–anchor consistency check, while deterministic model-transition replay detected ordering, artifact, and model-contract perturbations. Invalid signatures and mismatched DP bindings were rejected before aggregation; tampered copied DP records were detected through digest mismatch; commitment roots and public-input vectors were reconstructable; and the compact publication runs verified all submitted RSU/GLOBAL proofs. These results support artifact-level accountability, but they do not establish honest local training, raw-data provenance, runtime DP correctness, semantic correctness of learning, full poisoning resistance, or Sybil resistance without external enrollment governance.

The runtime results show that learning dominates the off-chain cost when the dataset is large and wide, as in CSE-CIC-IDS2018. When the learning core is smaller, as in CICIoV2024, commitment and proof costs become more visible. DP-record evidence was negligible in absolute time, while commitment construction and proof generation/verification were the main non-learning overheads. The on-chain results further show that receipt publication has a narrow and predictable role: it anchors accepted proof/public-input outcomes and digest pins without exposing raw telemetry or model updates.

The controlled federated stress experiment further addresses the operational assumptions of the compact benchmark. Under joint local-data-volume, label, and feature heterogeneity together with persistent, isolated, and burst contribution unavailability, 142 of 160 scheduled fits completed and the final GLOBAL model achieved 0.998151 accuracy, 0.984782 F1-score, and 0.998544 ROC-AUC. The stale-round and cross-RSU signed-context challenges were both rejected, while valid contributions continued to be admitted and aggregated. These results provide direct controlled FL-level evidence of successful operation under joint data heterogeneity and unstable participation, beyond the balanced-shard and stable-participation assumptions of the compact benchmark, without reinterpreting topology scaling as deployment validation.

Finally, the expanded-topology evaluation increased the hierarchy from 2 RSUs and 4 vehicles to 10 RSUs and 200 vehicles, and increased the submitted proof scopes from 5 to 21 per dataset. These results characterize structural scaling of the proof, export, and receipt-publication workflow rather than predictive invariance or deployment validity under larger and more heterogeneous IoV environments.

## 5. Limitations and Future Work

The reported evaluation supports FL-BC-IDS under a controlled prototype setting, but several limitations define the boundary of the claims. First, the verification workflow remains deliberately decomposed rather than encoding the complete learning program inside the Groth16 circuit. The workflow independently checks the model–anchor derivation relation and deterministically replays the recorded RSU model transition, while Groth16 verifies the scoped anchor-aggregation arithmetic. These checks establish consistency between the retained model artifacts and proof-oriented evidence under the declared deterministic implementation contracts. They do not prove the honest local training, raw-data provenance, correct optimizer execution, correct runtime DP execution, benign model updates, poisoning or backdoor absence, or full semantic correctness of the learned IDS model.

Second, the evaluation used a software-based hierarchical IoV prototype rather than a live V2X deployment. The compact full-system runs retain fixed train/validation/test artifacts, balanced training partitions, stable participation, two RSUs, four vehicles, and two federated rounds per RSU. The additional controlled stress evaluation extends this setting to 20 vehicles and eight rounds and explicitly introduces joint local-data-volume, label, and feature heterogeneity together with persistent, isolated, and burst contribution unavailability. It also exercises stale-round and cross-RSU signed-context admission challenges. Predictor-pattern-disjoint, supervised rolling-origin temporal, strict held-out-attack, and multiclass experiments provide complementary predictive-generalization evidence. The temporal evaluation remains a supervised periodic-refresh scenario and assumes that labels from the adaptation portion of the preceding interval become available before evaluation of the subsequent interval.

The controlled federated stress experiment remains an FL-level evaluation rather than a packet-level V2X network or physical-mobility simulation. Round-level contribution unavailability does not reproduce radio propagation or arbitrary communication delay, the stale-round challenge does not establish asynchronous-learning robustness under arbitrarily delayed model updates, and cross-RSU scope rejection does not establish stateful seamless handoff. The evaluated dataset representations do not provide the physical vehicle trajectories, radio-coverage/association traces, or source-vehicle state needed for those claims. Accordingly, physical mobility, time-varying RSU association, wireless link dynamics, and seamless handoff remain outside the validated deployment scope.

Third, the security guarantees remain bounded. Identity-bound admission, digest-bound DP records, deterministic commitments, Groth16 verification, and receipt publication improve accountability and auditability, but they do not provide complete poisoning resistance, Byzantine-robust learning, Sybil prevention, endpoint security, correct participant-key provisioning or revocation, or protection after private-key compromise. Similarly, the blockchain layer anchors verification outcomes and digest pins; it does not execute training, inspect raw data, recompute full artifacts, or guarantee that upstream operational decisions were fair.

Fourth, the numerical differential-privacy budgets reported in this study are conditional learner-stage guarantees for learner-input record instances, not end-to-end guarantees for original records. The evaluated preprocessing fits imputation, scaling, encoding parameters when applicable, and feature-domain bounds from the training partition, and train-only oversampling may replicate an original record. These operations were not assigned a separate DP preprocessing budget or a guaranteed original-record multiplicity cap. End-to-end original-record accounting therefore requires preprocessing quantities fixed independently of the protected records, for example from public or externally specified sources, or quantities obtained through separately accounted differentially private estimation, together with bounded preprocessing multiplicity and the composition defined in Equations (20) and (21).

Finally, the expanded 10-RSU/200-vehicle evaluation characterizes structural scaling of the proof, export, and publication workflow only; it is not interpreted as evidence of predictive invariance or deployment validity under larger IoV populations. Future work should therefore extend the controlled FL-level stress evaluation to live or high-fidelity V2X environments with physical vehicle mobility, time-varying RSU association, packet-level wireless loss and delay, stateful seamless handoff, larger stochastic participation changes, and cross-layer sensing–communication–computing interactions [2]; add stronger poisoning, Byzantine, Sybil, and fairness defenses; evaluate public or differentially private preprocessing contracts with bounded record-level resampling and end-to-end original-record accounting; develop richer proof semantics for local processing; optimize commitment/proof generation and receipt publication; and extend the supervised temporal-refresh evaluation toward delayed-label and unsupervised drift adaptation, explicit novelty and open-set detection, partition-aware tuning, feature engineering, dataset-specific DP tuning, knowledge distillation, and additional model families [17].

## 6. Conclusions

This paper presented FL-BC-IDS, an evidence-native privacy-aware hierarchical federated intrusion-detection framework for the Internet of Vehicles. The framework keeps raw vehicular telemetry local, trains vehicle-side DP-XGBoost models, performs deterministic RSU admission and aggregation, combines validated RSU outputs at the GLOBAL stage, and exports evidence that makes selected post-run properties independently checkable. The evidence path combines DP records, model-update digests, Ed25519 signed reports, deterministic model–anchor consistency checks, model-transition replay, SHA-256 and Poseidon commitments, Groth16 proof artifacts, reconstructable public inputs, and blockchain receipt pins within one audit-oriented workflow.

The compact full-system runs showed high predictive utility on the controlled principal benchmark partitions. Across the 10 seed-controlled runs, FL-BC-IDS achieved 0.998021±0.000246 accuracy, 0.983597±0.002053 F1-score, and 0.999272±0.000334 ROC-AUC on CSE-CIC-IDS2018, and 0.999867±0.000152 accuracy, 0.999495±0.000579 F1-score, and 0.999996±0.000005 ROC-AUC on CICIoV2024.

The supervised rolling-origin temporal evaluation on CSE-CIC-IDS2018 achieved a pooled seen-attack recall of 0.984788 at a pooled test FPR of 0.005700 across 6968 temporally shifted attack observations. Among the 6320 observations whose predictor hashes were novel relative to the corresponding training history, recall remained 0.983228. Under the strict predictor-hash-disjoint leave-one-attack-out protocol with development-only operating-point calibration, the macro-averaged held-out-attack recall was 0.8031 on CSE-CIC-IDS2018 and 0.9090 on CICIoV2024. Predictor-pattern-disjoint and multiclass experiments provided additional complementary generalization evidence.

A separate 20-vehicle, 8-round CSE-CIC-IDS2018 federated stress evaluation introduced joint local-data-volume, label, and feature heterogeneity together with persistent, isolated, and burst contribution unavailability. Across 160 scheduled fit opportunities, 142 completed, corresponding to an observed participation rate of 0.8875. The final GLOBAL model achieved 0.998151 accuracy, 0.983990 recall, 0.984782 F1-score, and 0.998544 ROC-AUC, while the stale-round and cross-RSU signed-context challenges were rejected at admission and the materialized RSU/GLOBAL proof scopes verified successfully.

The 20-round CSE-CIC-IDS2018 run demonstrated continued RSU-level federated operation beyond the two-round compact configuration, while the 10-RSU/200-vehicle runs increased the exercised proof scopes from 5 to 21 per dataset and characterized proof, export, and receipt-publication behavior under a larger hierarchy.

The evidence results support the main accountability claim: invalid signatures and DP-binding mismatches were rejected, copied DP-record tampering was detected, commitment values and public inputs were independently reconstructable, and all compact proof scopes were verified and receipt-pinned without exposing raw telemetry or model updates on-chain. A separate controlled verification additionally demonstrated deterministic model–anchor consistency checking and model-transition replay, including rejection of the valid-signature/unrelated-anchor counterexample. FL-BC-IDS does not eliminate every trust assumption or claim universal IoV security. Rather, its contribution is narrower and more defensible: it reduces blind trust in completed hierarchical FL runs by making selected participation, privacy-record, commitment, proof, and receipt outcomes reviewable from retained artifacts and public blockchain receipts.

## Figures and Tables

**Table 1:** Comparison of directly related blockchain-assisted and verifiable federated IDS frameworks across the evidence-workflow dimensions evaluated in this paper.

Work	Core Mechanism Focus	Declared Privacy Evidence	Identity-Bound Admission Evidence	Canonical Exported Artifacts	Independent Auditor Recomputation	ZK/Verifiable Computation	Receipt-Grade On-Chain Anchoring
Liu et al. [18]	Masked uploads, RSU pre-aggregation, and trust-driven model-chain consensus	∼	∼	✗	✗	✗	✗
Abdel-Basset et al. [19]	Blockchain-managed transformer IDS with miner-side gradient screening	∼	∼	✗	✗	✗	✗
Lv et al. [20]	Gaussian-DP misbehavior detection with accuracy-driven blockchain consensus	∼	∼	✗	✗	✗	✗
Jiang et al. [21]	Adaptive-DP federated data sharing with Delegated Byzantine Fault Tolerance (DBFT)-integrated update screening	∼	∼	✗	✗	✗	✗
Xie et al. [23]	Dual-chain model/log recording with InterPlanetary File System (IPFS)-backed storage and encrypted logs	∼	✗	✗	✗	✗	✗
Abou El Houda et al. [24]	Subjective-logic reputation ledger for participant trust management	✗	✗	✗	✗	✗	✗
Ullah et al. [25]	Blockchain logging of selected/aggregated models with top-*K* score-based selection	✗	✗	✗	✗	✗	✗
Smahi et al. [26]	Consortium-chain FL with zk-SNARK-style verification of local federated support vector machine (SVM) model claims	∼	∼	∼	✗	✓	✗
Wang et al. [22]	Consortium-chain FL with homomorphic encryption (HE)-protected updates and ciphertext-level robust filtering	∼	∼	✗	✗	✗	✗
**Proposed FL-BC-IDS**	Identity-bound participation, declared learner-stage DP evidence, canonical per-client envelopes, dual Merkle commitments, scoped Groth16 verification, and public receipt anchoring	✓	✓	✓	✓	✓	✓

**Table 2:** Security and verification scope of FL-BC-IDS.

Aspect or Attack Class	Status	Interpretation in This Paper
Declared learner-stage DP evidence and digest binding	✓	DP records persist the conditional learner-stage privacy account and are bound into downstream artifacts; they neither prove correct runtime DP execution nor establish end-to-end original-record differential privacy.
Identity-bound participation	✓	Admitted updates are signature-verified and tied to stable public-key-derived identifiers, with explicit admission or exclusion outcomes.
Canonical artifact integrity	✓	Canonical encodings, hashes, manifests, and commitments support artifact-level recomputation and tamper detection.
Scoped Groth16 verification	✓	Groth16 verifies the declared anchor-aggregation-consistency statement. Model–anchor derivation consistency and the recorded RSU model transition are checked separately by deterministic reconstruction and aggregation replay; Groth16 itself does not prove honest local training or full learning correctness.
On-chain receipt publication	✓	The chain records verification outcomes and fixed-length artifact pins, not raw telemetry, model updates, or per-client records.
Rejected before admission	✓	Malformed signatures, invalid round or RSU-scope binding, and mismatched contribution metadata are excluded before aggregation.
Detected or independently checkable	✓	Artifact substitution, digest mismatch, Merkle-root mismatch, public-input disagreement, and receipt-pin inconsistency are exposed by auditor recomputation or receipt cross-checking.
Poisoning, backdoors, collusion, Sybil, and Byzantine or malicious aggregation	✗	Identity binding and explicit evidence improve accountability, but the framework does not provide semantic poisoning or backdoor detection, collusion resistance, Byzantine-robust aggregation, malicious-aggregation resistance, or complete Sybil prevention.
Honest training, raw-data provenance, and endpoint or signing-key security	✗	The framework does not prove honest local training, verified raw-data provenance, uncompromised endpoints or signing keys, or correct runtime DP execution.

**Table 3:** Preprocessed evaluation artifacts. Training counts are reported after train-only imbalance correction; validation and test counts retain their pre-correction stratified-partition cardinalities.

Dataset	Training Artifact	Validation	Test	Features	Attack Surface
CSE-CIC-IDS2018	3,944,668	450,000	450,001	85 numeric	External network-flow IDS
CICIoV2024	1,713,232	211,233	211,233	9 numeric	Internal CAN-bus IDS

**Table 4:** Main split separation properties and generalization-validation protocols.

Dataset	Main Split	Time/Session/Source-Vehicle Separation	Exact Predictor-Pattern Disjointness in Main Split	Generalization Validation
CSE-CIC-IDS2018	Deterministic stratified row-level 70/15/15	Main split is not chronological or Flow-ID-disjoint; timestamp and Flow ID permit additional tests, but Flow ID is used only as a dataset-provided grouping proxy and no suitable source-vehicle identifier is available.	No; 1376/450,001 test rows (0.306%) match a training predictor vector.	Exact-pattern-group-disjoint binary, Flow-ID-disjoint binary, supervised rolling-origin temporal-refresh evaluation with development-only operating-point calibration, strict predictor-hash-disjoint leave-one-attack-type-out evaluation with development-only calibration, and nine-class recognition.
CICIoV2024	Deterministic stratified row-level 70/15/15	The selected decimal representation provides no timestamp, session identifier, or source-vehicle identifier.	No; 210,792/211,233 test rows (99.791%) match a training predictor vector.	Pattern-disjoint leave-one-attack-family-out and strict pattern-group-disjoint six-class recognition.

**Table 5:** Main experimental configurations.

Configuration	Purpose and Main Settings
Full FL-BC-IDS compact runs	2 RSUs, 4 vehicles, 2 federated rounds per RSU, 10 local boosting iterations per vehicle per federated round, DP-XGBoost enabled, evidence export, Groth16 verification, and receipt publication.
Matched non-DP hierarchical references	Same compact hierarchy and learning workflow, but DP disabled. Used to isolate the utility effect of the DP-enabled learner under the shared preprocessing contract.
Centralized XGBoost baselines	Single centralized non-FL XGBoost model trained on the full training split. Used as a predictive-utility reference.
Compact security-evidence checks	Same compact topology, with controlled invalid-signature, DP-binding, tamper, commitment-recomputation, and public-input agreement checks.
Controlled model-linkage and aggregation-replay validation	Standalone verification-layer evaluation of signed model-update binding, model–anchor reconstruction, deterministic model-transition replay, artifact substitution, contribution-order modification, and XGBoost model-contract mismatch. This validation does not retrain or alter the predictive-learning experiments.
Expanded evidence-workflow scale runs	10 RSUs and 200 vehicles with enlarged proof capacities. Used to characterize growth in proof scopes, artifact export, and receipt-publication cost under a larger hierarchy; this configuration is not treated as a deployment-validity or predictive-scaling endpoint.
Controlled heterogeneity and participation stress	CSE-CIC-IDS2018; 2 RSUs, 20 vehicles, 8 federated rounds, 10 local boosting iterations per vehicle per participating round, and seed 42. The condition combines heterogeneous local data volumes, label proportions, and feature distributions with persistent, isolated, and burst contribution unavailability. One stale-round signed-context challenge and one cross-RSU signed-context challenge are additionally exercised at admission. Validation and test artifacts remain unchanged.
20-round federated run	2 RSUs, 4 vehicles, 20 federated rounds per RSU, and 10 local boosting iterations per vehicle per federated round on CSE-CIC-IDS2018. Used to characterize longer-horizon operation of the privacy-aware hierarchical workflow beyond the compact two-round configuration.

**Table 6:** Representative seed-42 GLOBAL ensemble test performance of FL-BC-IDS on the compact full-system runs.

Dataset	Acc.	Prec.	Rec.	F1	ROC-AUC	Log-Loss	FP	FN
CSE-CIC-IDS2018	0.998138	0.991986	0.977264	0.984570	0.999052	0.033335	216	622
CICIoV2024	0.999711	0.997944	0.999855	0.998899	0.999920	0.025277	57	4

**Table 7:** Summary of the dataset-generalization stress tests.

Dataset	Protocol	Held-Out Result
CSE-CIC-IDS2018	Exact predictor-hash-group-disjoint binary	Accuracy 0.999718; recall 0.997744; F1 0.997690; ROC-AUC 0.999959.
CSE-CIC-IDS2018	Flow-ID-disjoint binary	Accuracy 0.999705; recall 0.997529; F1 0.997583; ROC-AUC 0.999973.
CSE-CIC-IDS2018	Supervised rolling-origin temporal refresh	Across eligible chronological transitions, pooled seen-attack recall was 0.984788 at a pooled test FPR of 0.005700 over 6,968 temporally shifted attack observations; recall remained 0.983228 across 6320 predictor-hash-novel observations.
CSE-CIC-IDS2018	Nine-class predictor-hash-group-disjoint	Accuracy 0.991180; balanced accuracy 0.994269; macro-F1 0.943428.
CICIoV2024	Six-class strict predictor-hash-group-disjoint train/test	Accuracy 0.999779; balanced accuracy 0.999955; macro-F1 0.999246; train–test predictor-hash overlap 0.

**Table 8:** Summary of the strict leave-one-attack-out evaluation with development-only operating-point calibration. Each held-out attack was absent from model fitting and calibration, predictor-hash overlap across the evaluated partitions was zero, and the primary threshold was fixed using a 1% benign development-FPR criterion.

Dataset	Held-Out Attacks	Macro Recall	Median Recall
CSE-CIC-IDS2018	7	0.8031	1.0000
CICIoV2024	5	0.9090	0.999946

**Table 9:** Controlled CSE-CIC-IDS2018 federated stress evaluation under joint data heterogeneity and unstable contribution availability.

Stress Dimension	Observed Condition or Outcome
Topology and training	20 vehicles, 2 RSUs, 8 federated rounds, and 10 local boosting iterations per participating vehicle per round.
Local data volume	60,353–433,518 samples per client; 7.18× maximum-to-minimum ratio.
Label heterogeneity	Local attack fractions ranged from 0.00879 to 0.87028.
Feature heterogeneity	Contiguous within-class value bands of a deterministically selected high-variance training feature were allocated across clients without altering the underlying feature values.
Contribution availability	142 of 160 scheduled vehicle-round fits completed; 18 were intentionally unavailable; observed participation rate 0.8875.
Signed-context admission	1/1 stale-round challenge rejected; 1/1 cross-RSU-scope challenge rejected.
Joint non-IID and participation-stress outcome	Under the simultaneous quantity, label, and feature heterogeneity and unstable participation conditions above, the final GLOBAL model achieved accuracy 0.998151, precision 0.985575, recall 0.983990, F1-score 0.984782, and ROC-AUC 0.998544 on the unchanged test set.
Evidence verification	Materialized round-6 challenge and round-8 terminal RSU anchor-aggregation proof scopes verified successfully, as did the final GLOBAL proof. Independent commitment recomputation and authoritative public-input reconstruction passed for the configured terminal audit scope.

**Table 10:** Representative seed-42 controlled design-reference comparison for isolating DP-learner and distributed-learning effects within the FL-BC-IDS experimental contract.

Dataset	Configuration	Acc.	Prec.	Rec.	F1	ROC-AUC	FP/FN	Comparison Meaning
CSE-CIC-IDS2018	Full FL-BC-IDS	0.998138	0.991986	0.977264	0.984570	0.999052	216/622	DP-enabled learner and complete evidence workflow retained.
CSE-CIC-IDS2018	Non-DP GLOBAL reference	0.999680	0.997951	0.996783	0.997367	0.999986	56/88	DP learner mode and declared DP-evidence binding removed.
CSE-CIC-IDS2018	Centralized XGBoost	0.999647	0.999339	0.994846	0.997088	0.999945	18/141	FL partitioning, raw-data locality, RSU/GLOBAL aggregation, DP, proof generation, and publication removed.
CICIoV2024	Full FL-BC-IDS	0.999711	0.997944	0.999855	0.998899	0.999920	57/4	DP-enabled learner and complete evidence workflow retained.
CICIoV2024	Non-DP GLOBAL reference	0.999981	1.000000	0.999855	0.999928	1.000000	0/4	DP learner mode and declared DP-evidence binding removed.
CICIoV2024	Centralized XGBoost	0.999991	1.000000	0.999928	0.999964	1.000000	0/2	FL partitioning, raw-data locality, RSU/GLOBAL aggregation, DP, proof generation, and publication removed.

**Table 11:** Multi-seed statistical validation of the principal learning configurations across 10 seed-controlled runs. Values are reported as mean ± standard deviation with 95% confidence intervals.

Metric	Centralized	Non-DP FL	FL-BC-IDS
CSE-CIC-IDS2018			
Accuracy	0.999630 ± 0.000032 [0.999611, 0.999648]	0.999360 ± 0.000093 [0.999307, 0.999415]	0.998021 ± 0.000246 [0.997884, 0.998170]
Precision	0.998487 ± 0.000773 [0.998020, 0.998921]	0.995858 ± 0.001687 [0.994845, 0.996808]	0.991146 ± 0.005059 [0.988044, 0.993976]
Recall	0.995419 ± 0.001170 [0.994749, 0.996118]	0.993613 ± 0.001186 [0.992918, 0.994308]	0.976206 ± 0.005646 [0.972933, 0.979526]
F1-score	0.996949 ± 0.000264 [0.996790, 0.997098]	0.994732 ± 0.000763 [0.994292, 0.995183]	0.983597 ± 0.002053 [0.982440, 0.984836]
ROC-AUC	0.999920 ± 0.000028 [0.999903, 0.999936]	0.999735 ± 0.000111 [0.999664, 0.999794]	0.999272 ± 0.000334 [0.999054, 0.999439]
CICIoV2024			
Accuracy	0.999989 ± 0.000005 [0.999986, 0.999992]	0.999987 ± 0.000005 [0.999984, 0.999990]	0.999867 ± 0.000152 [0.999772, 0.999952]
Precision	0.999989 ± 0.000024 [0.999975, 1.000000]	1.000000 ± 0.000000 [1.000000, 1.000000]	0.999077 ± 0.001146 [0.998360, 0.999715]
Recall	0.999928 ± 0.000038 [0.999906, 0.999949]	0.999902 ± 0.000042 [0.999877, 0.999924]	0.999913 ± 0.000049 [0.999884, 0.999942]
F1-score	0.999958 ± 0.000021 [0.999946, 0.999971]	0.999951 ± 0.000021 [0.999939, 0.999962]	0.999495 ± 0.000579 [0.999132, 0.999816]
ROC-AUC	1.000000 ± 0.000000 [1.000000, 1.000000]	1.000000 ± 0.000000 [1.000000, 1.000000]	0.999996 ± 0.000005 [0.999993, 0.999998]

**Table 12:** Proof-verification and receipt-publication outcomes for the compact full-system runs.

Evidence Item	CSE-CIC-IDS2018	CICIoV2024	Interpretation
RSU-scope proofs	4	4	One proof per RSU-round scope in the main run.
GLOBAL-scope proof	1	1	One final GLOBAL proof for the retained run-level aggregation scope.
Submitted proofs	5	5	Main compact run: 2 RSUs × 2 rounds + 1 GLOBAL proof.
Proof-verification outcome	All successful	All successful	No evaluated RSU/GLOBAL proof failed.
Exported bundle and receipt records	Generated	Generated	Artifacts were available for recomputation, audit, and receipt cross-checking.

**Table 13:** Controlled security-evidence checks across compact CSE-CIC-IDS2018 and CICIoV2024 runs.

Check	Manipulation or Verification Action	Recorded Outcome	CSE-CIC Outcome	CICIoV2024 Outcome
Bad-signature exclusion	Corrupted Ed25519 signature in a signed vehicle report	Invalid signed participation evidence was rejected before aggregation.	Rejected	Rejected
Bad DP-binding exclusion	Mismatched DP-record binding	The participant was excluded with an explicit digest-binding failure classification.	Excluded	Excluded
DP-record tamper detection	Modified copied DP record after export	Digest mismatch detected the tampered DP record non-destructively.	Detected	Detected
Commitment recomputation	Recomputed RSU and GLOBAL commitment roots	Recomputed SHA-256/Poseidon roots and GLOBAL manifest bindings matched the exported artifacts.	Matched	Matched
Public-input agreement	Standalone public-input vector reconstruction	The expected 10-element public-input vector matched the exported vector exactly.	Matched	Matched

**Table 14:** Controlled model-linkage and deterministic aggregation-replay validation.

Validation Case	Expected Verification Behavior	Outcome
Valid model–anchor relation	The controlled serialized model contribution and corresponding anchor satisfy deterministic reconstruction and digest checks.	Passed
Valid signature with unrelated anchor	The signature remains valid, but an anchor associated with a different model contribution must fail model–anchor consistency verification.	Rejected
Signed model-update substitution	Received model bytes differing from the signed update digest must fail before model reconstruction.	Rejected
Signed anchor-vector substitution	An altered anchor vector retaining the original signed anchor digest must fail the anchor-integrity check.	Rejected
Deterministic model reconstruction	Reconstruction from the controlled pre-update model state and serialized contribution must reproduce the corresponding post-update model behavior.	Passed
Deterministic aggregation replay	Repeated execution from identical ordered controlled inputs must reproduce the same aggregate transition.	Passed
Contribution-order modification	Changing the recorded admitted-contribution order must be detectable by the replayed transition.	Detected
Aggregate-artifact modification	Modification of the retained aggregate artifact must produce a digest mismatch.	Detected
Incompatible XGBoost contract	Model artifacts with incompatible XGBoost contracts must be rejected rather than replayed as one transition.	Rejected
Subsequent federated transition	The replay procedure must remain applicable to a later model transition, not only the initial aggregation scope.	Passed

**Table 15:** Recorded Sepolia publication outcomes for the compact FL-BC-IDS workflow, including proof verification, publication cost, transaction latency, and consensus finality.

Aspect	CSE-CIC-IDS2018	CICIoV2024
Submitted proofs	5	5
Verified proofs	5	5
Calldata volume	4116 bytes	4116 bytes
Total gas used	4,653,922	4,653,874
Total ETH spent	0.009307844054	0.009307748060
Run finalization	Successful	Successful
**Instrumented transaction and consensus-finality measurements**		
Proof-publication transactions	5	
Successful proof transactions	5/5	
Observed proof-transaction failures	0/5 (0%)	
Successful Groth16 verification outcomes	5/5	
Observed proof-verification failures	0/5 (0%)	
Proof-transaction total latency	Mean 13.685 s; median 11.758 s; p95 21.917 s	
Receipt/inclusion wait latency	Mean 11.972 s; median 10.098 s; p95 20.163 s	
Transactions monitored for consensus finality	6/6 finalized (5 proof transactions + 1 run-finalization transaction)	
Proof receipt-to-finality latency	Mean 1093.514 s (18.23 min); median 1093.134 s; p95 1117.961 s	
Proof transaction-start-to-finality latency	Mean 1107.199 s (18.45 min); median 1104.498 s; p95 1139.878 s	
Finality timeouts	0/6	
Submission pattern	Serial single-sender publication with at most one transaction in flight; concurrent multi-sender submission was not evaluated	

**Table 16:** Coarse runtime decomposition of the compact full-system runs.

Dataset	Client Wall Time (s)	Local Training (s)	RSU Round Latency (s)	GLOBAL Agg. + Proof + Export (s)
CSE-CIC-IDS2018	270.546	262.124	18.301	0.86553
CICIoV2024	40.555	35.833	19.930	1.02225

**Table 17:** Layered off-chain overhead in the compact security-evidence runs.

Layer	CSE-CIC-IDS2018 (s)	CICIoV2024 (s)
Learning core	252.3369	34.4951
DP evidence	0.0373	0.0369
Identity-bound admission	2.5906	2.9588
Commitment construction	12.9838	14.3384
Proof generation/verification	8.8836	9.4173
Artifact export	0.0235	0.0247
Total compact run	276.8557	61.2712

**Table 18:** Evidence-workflow scaling from the compact to the expanded hierarchical configuration.

Dataset	Topology	Proof Scopes	Total Gas	Total ETH
CSE-CIC-IDS2018	2 RSUs/4 vehicles	5	4,653,922	0.009307844054
CSE-CIC-IDS2018	10 RSUs/200 vehicles	21	13,391,338	0.026782676271
CICIoV2024	2 RSUs/4 vehicles	5	4,653,874	0.009307748060
CICIoV2024	10 RSUs/200 vehicles	21	13,391,290	0.026782580277

Note: The compact topology uses 2 RSUs and 4 vehicles, whereas the expanded topology uses 10 RSUs and 200 vehicles. The proof count follows the submitted proof scopes: 2 RSUs × 2 rounds + 1 GLOBAL proof in the compact runs, and 10 RSUs × 2 rounds + 1 GLOBAL proof in the expanded runs.

## Data Availability

The datasets analyzed in this study are openly available from the Canadian Institute for Cybersecurity (CIC), University of New Brunswick: CSE-CIC-IDS2018 at https://www.unb.ca/cic/datasets/ids-2018.html and CICIoV2024 at https://www.unb.ca/cic/datasets/iov-dataset-2024.html (both accessed on 7 July 2026). The complete accepted-version reproducibility and verification package supporting this study is publicly available in the FL-BC-IDS GitHub repository at https://github.com/wisamalwashYildiz/FL-BC-IDS, tag v1.0.0 (commit 1822133a1d42d5a13cadb8f50694e85969f1235d; accessed on 24 August 2026). The public package includes the FL-BC-IDS source code, Groth16 circuit sources and verification materials, Solidity contracts, deterministic preprocessing and partition-reconstruction materials, experimental configurations and random seeds, retained evidence bundles, proof and public-input artifacts, auditor verification scripts, blockchain-publication records, selected execution logs, and executable verification instructions. The underlying benchmark datasets continue to be obtained from their authoritative sources rather than redistributed. Operational credentials, API tokens, private keys, wallet credentials, machine-specific local paths, and other security-sensitive deployment information are not research artifacts and are not included in the public repository.

## Abbreviations

Acc.	Accuracy
AMD64	64-bit x86 Architecture
API	Application Programming Interface
approxDP	DP-XGBoost Differentially Private Approximate Tree-Learning Mode
BN254	Barreto–Naehrig 254-bit Pairing-Friendly Curve
CAN	Controller Area Network
CIC	Canadian Institute for Cybersecurity
CICIoV2024	CIC Internet of Vehicles 2024 dataset
CPython	Reference Python implementation written in C
CSE-CIC	Communications Security Establishment–Canadian Institute for Cybersecurity
CSE-CIC-IDS2018	CSE-CIC Intrusion Detection System 2018 dataset
DBFT	Delegated Byzantine Fault Tolerance
DP	Differential Privacy
DP-XGBoost	Differentially Private XGBoost
ECE	Expected Calibration Error
ETH	Ether
F1	F1-score
FL	Federated Learning
FL-BC-IDS	Federated Learning–Blockchain Intrusion Detection System
FN	False Negative
FP	False Positive
GLOBAL	Global Prediction-Ensemble and Verification Stage
HE	Homomorphic Encryption
IDS	Intrusion Detection System
IoV	Internet of Vehicles
IPFS	Interplanetary File System
non-DP	Without Differential Privacy
non-FL	Without Federated Learning
non-IID	Not Independent And Identically Distributed
npm	Node Package Manager
Prec.	Precision
QKD	Quantum Key Distribution
Rec.	Recall
ROC-AUC	Area Under the Receiver Operating Characteristic Curve
SHA-256	Secure Hash Algorithm 256-bit
RSU	Roadside Unit
SVM	Support Vector Machine
TN	True Negative
TP	True Positive
V2X	Vehicle-to-Everything
XGBoost	Extreme Gradient Boosting
ZK	Zero-Knowledge
zk-SNARK	Zero-Knowledge Succinct Non-Interactive Argument of Knowledge
